# Clarifying Exercise Intolerance in *Cardiovascular* Disease: Protocol for a Scoping Review and Evolutionary Concept Analysis

**DOI:** 10.3390/nursrep16090323

**Published:** 2026-09-06

**Authors:** Joana Pereira Sousa, Paulo Santos-Costa, João Graveto, Paulo Ferreira, Maria Loureiro

**Affiliations:** University of Coimbra, Health Sciences Research Unit: Nursing (UICISA: E), Nursing School of the University of Coimbra, Rua 5 de Outubro, Apartado 7001, 3046-851 Coimbra, Portugal; paulocosta@ese.uc.pt (P.S.-C.); jgraveto@ese.uc.pt (J.G.); palex@ese.uc.pt (P.F.); marialoureiro@ese.uc.pt (M.L.)

**Keywords:** exercise intolerance, exercise tolerance, activity intolerance, cardiovascular diseases, Rodgers’ evolutionary concept analysis, scoping review, nursing

## Abstract

**Background/Objectives:** Exercise intolerance is clinically important but conceptually unstable in cardiovascular care. Its relationship with exercise tolerance, exercise capacity, functional capacity and the nursing concept of activity intolerance remains unclear, and the term entered nursing from other disciplines without corresponding conceptual scrutiny. This protocol will examine how these concepts are used in adults with cardiovascular disease, how use varies across conditions, care settings, pathway phases and time, and which attributes, antecedents, consequences and related concepts are reported, to determine what these concepts can mean for nursing. **Methods:** The study has two interconnected phases: a scoping review following Joanna Briggs Institute methodology and PRISMA-ScR and PRISMA-S reporting, followed by Rodgers’ evolutionary concept analysis. Searches will cover MEDLINE, CINAHL, Embase, Web of Science and Scopus, complemented by gray literature and standardized nursing terminologies. **Expected Results:** The review will map how exercise intolerance, activity intolerance and neighboring terms are used and will chart which defining attributes and empirical referents are recognizable through routine nursing assessment, require instrumented testing or both. Whether a stable nursing formulation can be derived is treated as an open question: convergence, divergence, instability and insufficient evidence are all possible, and attributes recognizable only through instrumented testing will be reported as a null result for nursing operationalization. **Conclusions:** The review will determine how the concept has evolved for the discipline of nursing and whether nursing assessment can operationalize it. It will not establish professional consensus, diagnostic thresholds, measurement validity or device specifications. **Registration:** This study was registered in the Open Science Framework (OSF) Registries before screening and data charting commenced.

## 1. Introduction

Cardiovascular diseases remain the leading cause of death worldwide and accounted for an estimated 19.8 million deaths in 2022 [1]. Cardiac rehabilitation, which combines patient assessment, aerobic and resistance exercise training, physical-activity counseling and risk-factor management, is a core secondary-prevention response [2]. Nurses assess and act on exertional tolerance throughout this pathway, including early mobilization after an acute event, education about pacing and warning symptoms, discharge planning and longer-term follow-up. However, the phenomenon assessed has no settled name. Nursing, cardiology, rehabilitation and exercise science use different terms, and exercise intensity is prescribed and evaluated on the basis of these terms [3,4].

For nursing, the problem has a further edge, because nursing carries a term of its own. Activity intolerance is a practice-oriented diagnostic label whose defining characteristics have been clinically validated in acute coronary syndrome, refractory angina and decompensated heart failure [5,6,7,8], and it is formally registered in the NANDA-International taxonomy [9] and in the International Classification for Nursing Practice [10]. Whether activity intolerance and exercise intolerance denote one concept, two related concepts or two context-specific formulations is unresolved. This review treats the question as open and does not describe them as the same phenomenon before analysis. The answer carries practical consequences, because what a nurse can recognize at the bedside, through symptoms, observed activity, perceived exertion and ordinary monitoring, is not necessarily what the cardiovascular literature uses to define the concept.

The difficulty is not that several terms exist, but that they are used as though interchangeable while denoting different things. Guazzi [11] and Brazile et al. [12] described cardiopulmonary exercise testing as a standard approach to the assessment of exercise intolerance, since it examines the integrated contribution of the pulmonary, cardiovascular, muscular and cellular oxidative systems and evaluates exertional symptoms, disease severity and prognosis. Within the same procedure, however, Sietsema and Rossiter [13] identified maximal oxygen uptake as the standard expression of aerobic capacity, so that a single test yields indicators belonging to different constructs, and the available interpretive recommendations address how those indicators are read rather than what they represent [14]. A comparable ambiguity affects field-based assessment, since Coulshed et al. [15] found the six-minute walk test useful for measuring and improving prognosis in ischemic heart disease, although the distance walked expresses everyday task performance rather than maximal physiological capacity. Cardiorespiratory fitness, by contrast, is not a test result but a physiological trait, characterized by Ross et al. [16] as the integrated ability of the circulatory and respiratory systems to supply oxygen during sustained physical activity. Exercise intolerance, in turn, is not simply the inverse of any of these. Del Buono et al. [17] described it in heart failure as a cardinal and multidimensional manifestation associated with cardiac reserve, pulmonary function, vascular function, skeletal muscle abnormalities, exertional symptoms, functional limitation, quality of life and prognosis. Prognostic value, moreover, does not establish conceptual identity, since Lang et al. [18] concluded that cardiorespiratory fitness is a strong and consistent predictor of morbidity and mortality without that association rendering it synonymous with exercise tolerance. Similarly, Nayor et al. [19] demonstrated that impaired exercise performance in heart failure with preserved ejection fraction reflects deficits across cardiac, pulmonary, vascular and skeletal muscle reserve rather than a single central abnormality. Consequently, a source reporting an exercise outcome may be describing maximal physiological capacity, achieved workload, everyday task performance, symptom-limited effort or recovery, and the term it uses does not reliably indicate which. Table 1 presents the provisional sensitizing descriptions adopted for these terms.

Clarifying the concept may contribute to nursing science and practice, because the gap concerning this phenomenon is disciplinary. Rodgers’ evolutionary method has been used in nursing science precisely to examine concepts whose meanings vary across settings and disciplines: critical thinking, where the analysis separated the disposition from the cognitive process and from the educational outcomes attributed to it [20], and transitional care, where it established the attributes that distinguish the concept from discharge planning and from continuity of care [21] and was subsequently examined in heart failure populations specifically [22]. Each of these analyses produced defining attributes, antecedents and consequences for the discipline and delimited the concept against its neighbors. None of them addressed exertional limitation, and the method has not been applied to exercise intolerance in any population. Within nursing, conceptual work on exertional limitation has been directed at activity intolerance [5,6,7,8,23], but always with that diagnostic label as the object of analysis. Exercise intolerance, by contrast, has entered nursing literature and practice from cardiology, rehabilitation and exercise physiology without corresponding conceptual scrutiny. Nursing, therefore, employs a term whose defining attributes, antecedents and consequences have not been established for its own domain, and whose relationship to the concept it does hold registered, activity intolerance [9,10], remains undetermined. What is missing is consequently an analysis of how exercise intolerance has evolved as a concept for the discipline of nursing, which of its attributes are recognizable and usable in nursing assessment, and whether it is distinct from, surrogate to or continuous with activity intolerance. Evidence produced in other disciplines is examined because that is where the term has largely developed, but it is examined to determine what the concept can mean for nursing knowledge and practice. The review does not seek to clarify the term for those disciplines.

This protocol, therefore, combines scoping review methodology, appropriate for examining heterogeneous terminology, definitions, evidence types and operational practices [24,25], with Rodgers’ evolutionary concept analysis, which treats concepts as dynamic, context-dependent phenomena and examines their uses, attributes, antecedents, consequences, surrogate terms and related concepts [26,27]. The review will yield a provisional conceptual synthesis only if the included literature supports one, and may instead demonstrate instability, competing meanings or insufficient evidence for a unified definition.

## 2. Materials and Methods

This study adopts a two-phase sequential design in which a scoping review provides the systematically retrieved evidence base for Rodgers’ evolutionary concept analysis. The scoping review will systematically identify, map and characterize the available literature concerning exercise intolerance in adults with cardiovascular disease, whereas the subsequent concept analysis will use the mapped evidence to examine how the concept has been used and how its meaning has evolved across clinical and temporal contexts, and what that evolution implies for nursing assessment.

The study will be conducted in two interconnected phases. In Phase I, a scoping review will be undertaken in accordance with the Joanna Briggs Institute (JBI) methodology for scoping reviews [24,25]. Reporting will follow the Preferred Reporting Items for Systematic Reviews and Meta-Analyses Extension for Scoping Reviews (PRISMA-ScR) [28], whereas the search process will additionally follow the PRISMA Search Reporting Extension (PRISMA-S [29]). The reporting of the search will follow PRISMA-P [30].

In Phase II, the evidence generated through the scoping review will be analyzed using Rodgers’ evolutionary method of concept analysis [26,27]. Rodgers’ approach conceptualizes concepts as dynamic, context-dependent phenomena that evolve through disciplinary knowledge, clinical practice and social context. Accordingly, the mapped literature will be examined to identify defining attributes, antecedents, consequences, surrogate terms, related concepts and contextual influences affecting the use of exercise intolerance across cardiovascular care.

### 2.1. Aim

To examine how exercise intolerance and its relationship with the nursing concept of activity intolerance are used in adults with cardiovascular disease, with attention to acute, post-acute, rehabilitation and longer-term recovery after cardiovascular events, by mapping variation across cardiovascular conditions, disciplines, care settings and time and applying Rodgers’ evolutionary concept analysis.

### 2.2. Review Questions

How are exercise intolerance and activity intolerance used, distinguished or related in the literature concerning adults with cardiovascular disease, particularly during recovery after a cardiovascular event?

#### Secondary Review Questions

Which terms function as surrogate terms for, or related concepts to, exercise intolerance and activity intolerance, and what evidence supports each classification?

Which defining attributes characterize exercise intolerance, and which attributes are specific to activity intolerance in nursing literature?

Which clinical, physiological, functional, psychological and contextual antecedents precede or influence exercise intolerance?

Which consequences are associated with exercise intolerance?

How are exercise intolerance, activity intolerance, exercise tolerance, exercise capacity, functional capacity, aerobic capacity and cardiorespiratory fitness distinguished or connected in the included literature?

Do included sources explicitly classify exercise intolerance into subtypes, or do they describe possible mechanisms or loci of limitation? Reviewer-inferred mechanisms will not be treated as subtypes unless the original source explicitly supports that classification.

Which observable indicators, defining attributes and empirical referents are recognizable through routine nursing assessment, require instrumented testing or require both, and what evidence supports their actionability in nursing practice? This is a charting question about conceptual referents, not a measurement-validity review; if the literature supports only instrumented referents, that will be reported as a null finding for point-of-care nursing operationalization.

Recognition and actionability will be assessed only where the source provides enough information to code them under the prespecified rules in Section 2.7, and no nursing interpretation will be used to override a negative finding.

Comparative dimensions are specified a priori. Care context will be classified by healthcare setting and phase of the cardiovascular pathway. The publication period will be analyzed using the prespecified cut-off of 2016/2017, anchored on the 2016 scientific statement on cardiorespiratory fitness as a clinical vital sign [16]. Cardiovascular condition will be coded using the expanded disease categories listed in Section 2.4, and disciplinary provenance recorded descriptively. This statement was preferred to adjacent cardiovascular publications of the same period, such as the 2013 scientific statement on exercise standards for testing and training [31] and the 2016 focused update on pharmacological therapy for heart failure [32], because it is the document that reframed an exercise-derived measure as a routine clinical vital sign and therefore marks the point at which exercise-related constructs entered general clinical assessment rather than remaining within exercise testing. The cut-off is a comparative device for examining temporal change, not a claim that conceptual usage altered abruptly in that year; sources on either side of it are treated identically at every other stage of the review.

### 2.3. Methodological Framework

This section specifies how the two phases described above are operationalized. Because methodological credibility depends on the relevance and quality of the evidence informing concept development, eligibility decisions will consider both conceptual contribution and source type, and, following Tofthagen and Fagerstrøm [27], the evidential status of included sources will be documented and weighed during interpretation as specified in Section 2.8.

Rodgers’ evolutionary method will be operationalized through six iterative analytical steps:

− Identify the focal concept and associated expressions.
− Define the disciplinary and clinical domains from which evidence will be sampled.
− Collect evidence describing contextual use, defining attributes, antecedents and consequences.
− Analyze patterns of convergence, divergence, contextual variation and temporal evolution.
− Construct an illustrative exemplar demonstrating the defining attributes of the concept.
− Identify implications, hypotheses and priorities for future concept refinement, operationalization and empirical validation.

The interface between the two phases is operationalized through the charting framework rather than left implicit. Each of Rodgers’ six steps is populated by named charting domains, and the step-by-step mapping is set out in full in Supplementary Table S2: terminology for step 1; provenance, temporal positioning, population and context for step 2; attributes, antecedents, consequences, operationalization and empirical referents for step 3; conceptual interpretation combined with the comparative dimensions prespecified in Section Secondary Review Questions for step 4; the attributes established in step 4 together with the recorded illustrative examples for step 5; and nursing recognizability, locus of limitation and charted evidential status for step 6. No step is populated from the reviewers’ recollection of the corpus: each is traceable to named fields in the charting form, so that the passage from retrieval to interpretation can be audited.

The scoping review will seek comprehensive retrieval and Rodgers’ analysis will interpret the full eligible corpus; no stopping rule will be applied to retrieval or eligibility. Should the corpus exceed what full charting and verification can accommodate within the registered timetable, the team will pause before charting, document the yield and submit a dated amendment specifying a transparent prioritization rule or a revised timetable, which is registered before charting begins; no post hoc convenience sample will be introduced silently. The estimated duration from the start of searching to submission of the completed review is fifteen to eighteen months, with the indicative milestone schedule registered in the Open Science Framework project.

### 2.4. Eligibility Criteria

Eligibility criteria were established a priori using the Population–Concept–Context (PCC) framework recommended by the Joanna Briggs Institute for scoping reviews [24,25]. The PCC framework (Table 2) was selected because it provides a structured approach for defining the scope of evidence while accommodating the conceptual breadth required for evolutionary concept analysis.

Based on this framework, studies will be eligible if they concern adults with cardiovascular disease and provide conceptual, definitional or interpretative information relevant to exercise intolerance, activity intolerance or a neighboring construct that is explicitly linked to one of these focal concepts. A source about exercise capacity, functional capacity, cardiorespiratory fitness or physical fitness will not be eligible solely because it reports an exercise outcome. It must define, distinguish, interpret or discuss the construct in relation to exercise intolerance or activity intolerance. Quantitative, qualitative and mixed-methods studies, reviews, clinical practice guidelines, professional position and consensus statements, theoretical and discussion papers, methodological publications, doctoral dissertations and master’s theses, and institutional reports will be considered eligible. Editorials, letters to the editor and commentaries are eligible only where they advance an explicit definitional or conceptual argument rather than opinion alone. Conference abstracts are not eligible because they rarely carry sufficient definitional detail to be charted across the framework and cannot be verified against a full text; where one signals a conceptually important study, the full report is sought under the procedure in Section 2.5.

No restrictions will be applied regarding healthcare setting or cardiovascular diagnosis. Acute, post-acute, rehabilitation and long-term recovery phases will be charted explicitly. Publications written in English, Portuguese and Spanish will be considered eligible; translation tools may support access to other languages only when a reviewer can verify the translation and the source is conceptually important. Database searches will run from inception to the final search date, with no temporal restrictions.

The bibliographic search will be conducted in five electronic databases selected for complementary coverage: MEDLINE via PubMed; CINAHL Complete via EBSCOhost; Embase via Ovid; Web of Science Core Collection; and Scopus. The disease block will include cardiovascular disease, heart disease, heart failure, coronary artery disease, coronary heart disease, myocardial infarction, ischemic heart disease, valvular heart disease, cardiomyopathies, atrial fibrillation and broader arrhythmias, hypertension, pulmonary hypertension, cerebrovascular disease, stroke, peripheral arterial disease, congenital heart disease, cardiac rehabilitation and relevant controlled vocabulary.

Gray literature will be searched in Google Scholar, ProQuest Dissertations & Theses Global and RCAAP. The first 200 Google Scholar records ranked by relevance will be screened for each documented search, while RCAAP records returned by the documented strategies will be screened in full. Backward and forward citation searching will be undertaken for included sources and conceptually informative reviews. Gray literature is eligible on the criteria stated above and is actively sought rather than accepted incidentally, because definitional and conceptual material frequently appears there before it reaches, or instead of reaching, the journal literature.

Because the relationship between activity intolerance and exercise intolerance is one of the review questions, the standardized nursing terminologies in which activity intolerance is formally registered are treated as information sources rather than as incidental gray literature. The diagnostic label, together with its definition, defining characteristics, related factors and associated conditions, will be retrieved from the NANDA-International taxonomy [9] and from the International Classification for Nursing Practice [10], in the editions current at the date of extraction, and the wording of each will be recorded verbatim in the charting form together with the diagnostic code, the edition and the date of access. Successive editions will be compared where they are accessible, since the label appears to have been revised during the period under review. Where the two terminologies differ in how they define or delimit the concept, the difference will be reported rather than reconciled, because divergence between formal nursing terminologies is itself evidence about the stability of the concept.

### 2.5. Search Strategy

The search strategy is built as four conceptual strings: (1) exercise intolerance and exercise tolerance; (2) activity intolerance and closely related nursing expressions; (3) cardiovascular disease; and (4) conceptual or terminological framing. The focal and nursing strings will be combined with the cardiovascular block and, where applicable, the conceptual block. The disease block is expanded across cardiovascular conditions and will be adapted with executable free-text syntax for every database. Conceptual terms include concept*, defin*, taxonom*, terminolog*, nomenclature and construct*. Measurement, determinant and predictor terms are not used as search restrictions. A master search strings is presented in Table 3.

The search strategies in Supplementary Table S1 are draft/planned strategies unless an execution date and retrieval yield are explicitly recorded. The initial MEDLINE feasibility search conducted on 31 May 2026 is reported separately from the planned review searches. For every database and interface, the final executable syntax, field codes, controlled vocabulary, search date, limits, yield, export and deduplication notes will be recorded after execution.

No context block will be incorporated into the search strategy because the review intentionally examines conceptual use across the entire cardiovascular care continuum.

Three syntax decisions are reported explicitly. Quoted phrases will not be truncated; single-term truncation will be used only where supported by the interface. The master syntax will be translated rather than transposed across MEDLINE, CINAHL, Embase, Web of Science and Scopus. Each adapted strategy will be reported in full, and no planned combination contains placeholders in place of executable syntax.

C3 is not conditional on the yield of C1. It is run in every database as a primary combination. C3 removes the conceptual-framing block (String 4) while retaining the focal-concept block (String 1), so that it retrieves records which name exercise intolerance or activity intolerance in the title or abstract but do not signal conceptual or definitional framing there—characteristically empirical papers whose definition or operationalization of the concept appears in the Methods or Results—rather than leaving them to a contingent sensitivity check. Records retrieved by C3 are screened by title and abstract. The number of records contributed by C3, and the number of C3 records ultimately included, will be reported separately for each database. Records retrieved by C3 enter the same two-stage screening as those retrieved by C1 and C2, dually and blinded, against the same criteria and with no relaxed threshold; the yields of C1 and C3 are compared descriptively per database; and a record identified only by C3 carries no additional requirement to display conceptual vocabulary. The three procedural rules are set out in full in Supplementary Table S1. Should the proportion of included sources unique to C3 prove substantial, that will be reported as evidence that conceptual framing vocabulary is an unreliable retrieval filter for this literature, which is itself a finding relevant to the argument of the review.

The PRESS-based peer review has not yet been carried out; it is scheduled to precede execution of the review searches. Each database strategy will undergo PRESS-based peer review by a suitably experienced reviewer or information specialist and will be tested against prespecified sentinel publications from nursing, cardiology, rehabilitation, exercise physiology and the expanded cardiovascular disease categories. Failure to retrieve a known eligible source will prompt documented revision and revalidation.

All five database searches and the gray-literature searches will be updated in November 2026, before final synthesis and manuscript submission. The initial MEDLINE (via PubMed) feasibility search, the first full searches and the update searches will be reported separately. Where a potentially eligible record is unobtainable in full text, or where its definition of the focal concept cannot be established from the published text, the corresponding author will be contacted once by e-mail and a reminder sent after two weeks; records that remain unresolved will be reported as such in the PRISMA-ScR flow diagram, and no unpublished definition will be inferred.

### 2.6. Study Selection

All retrieved records will be imported into Rayyan^®^ for reference management, duplicate removal and study selection. Screening will be conducted in two sequential stages: (1) title and abstract screening and (2) full-text assessment.

Before formal screening, all reviewers will independently assess a common pilot sample of at least 50 title and abstract records and at least 10 full-text articles to calibrate the eligibility criteria. Agreement will be quantified rather than described. Fleiss’ kappa will be computed across the four reviewers on the common pilot sample, and Cohen’s kappa for each operational reviewer pair, since screening proper is conducted in pairs. Formal screening will begin when every operational pair satisfies one of two prespecified conditions. The first is a Cohen’s kappa of at least 0.70. The second applies only where the small proportion of eligible records at this stage depresses kappa despite high observed agreement and requires all three: raw agreement of at least 0.90, a prevalence-adjusted bias-adjusted kappa of at least 0.70, and a written account attributing the residual disagreements to correctable ambiguities in the criteria. These are alternative routes to the same threshold of reliability, not an exception to it: a pair that satisfies neither does not begin screening, and the criteria and screening guidance are revised and a further pilot round conducted on a fresh sample. The number of calibration rounds, the statistics obtained at each and the resulting revisions will be reported.

Following calibration, records will be divided between two reviewer teams. Within each team, two reviewers will independently screen the assigned titles and abstracts and subsequently assess the corresponding full-text articles while remaining blinded to one another’s decisions within Rayyan. Disagreements will first be resolved through discussion and, when necessary, by consultation with a third reviewer.

**Figure 1:**
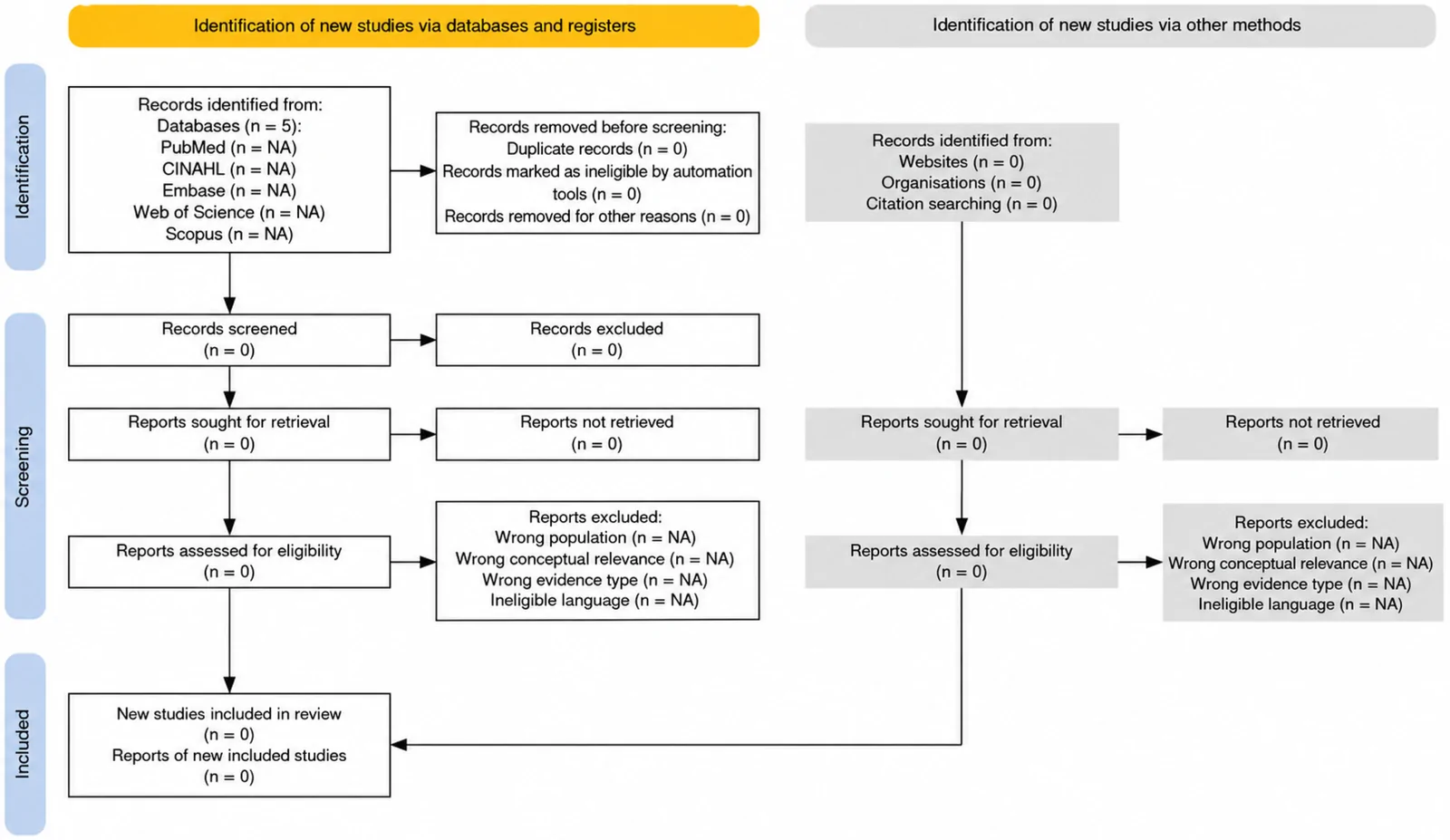
PRISMA-ScR flow diagram for the planned scoping review. All counts will be completed after the searches, screening and full-text assessment have been executed and will be reported separately for the original and the updated searches [33].

One primary reason for exclusion will be recorded for each study excluded after full-text assessment. The study selection process will be reported using the PRISMA-ScR flow diagram shown in Figure 1, documenting the number of records identified, screened, assessed for eligibility and included in the review.

The flow diagram will report the counts required by PRISMA-ScR at every stage, disaggregated by database and by gray-literature source; the reasons for exclusion at full text will be tabulated by category in a Supplementary Table and the corresponding record list deposited in the OSF project (https://doi.org/10.17605/OSF.IO/7XZ39).

### 2.7. Data Charting

Data will be extracted using a standardized charting form developed by the review team and pilot-tested before formal extraction. The form may be refined iteratively during the review, with all modifications documented and reported.

Data charting will record whether an included source explicitly identifies a locus or mechanism of limitation. Categories such as central/cardiac, peripheral/muscular, ventilatory and symptomatic/perceptual will be treated as source-reported mechanisms or classifications, not as presumed conceptual subtypes. Source-reported and reviewer-inferred mechanisms are held in two separate charting fields, as set out in Supplementary Table S2: the source-reported field records only what the source itself states and is coded as not stated where the source states none, while any mechanism the reviewer reads into a source is entered in the reviewer-inferred field, is never combined with the source-reported field in any count, and is never used to claim that the concept has subtypes. Each defining attribute and empirical referent will also be coded as recognizable through routine nursing assessment, through instrumented testing, or through both. The rule is applied to the way the source obtains the referent and not to the parameter itself: heart rate, blood pressure, peripheral oxygen saturation, rhythm read from routine monitoring, exertional symptoms and perceived exertion are coded as routine nursing assessment when obtained during usual care, and as instrumented testing where they exist only as products of a standardized protocol or of specialized acquisition, such as a chronotropic index, a twelve-lead exercise ECG, a heart rate variability index or a Borg value recorded at a prescribed workload. A referent the source supports by both routes is coded as both; one whose route the source does not state is coded as unclear and does not count towards nursing recognizability. The boundary is anchored to external referents rather than to reviewer judgment alone: the routine route is delimited by the defining characteristics of activity intolerance in the NANDA-International taxonomy and the corresponding International Classification for Nursing Practice concepts together with standard bedside assessment parameters, and the instrumented route by the procedures that require a laboratory, a standardized protocol, gas exchange analysis, a dedicated sensor or specialized ECG analysis. Both reference lists and the eleven worked examples that fix the rule’s application to each of these measures are settled before charting begins and reproduced in Supplementary Table S2, where they are binding on all four reviewers. Actionability is coded only where the source links a referent to a nursing action; otherwise it is recorded as recognizable but actionability not demonstrated. Disagreements are resolved through source-location review, pair discussion, codebook amendment and third-reviewer adjudication, with the decision and its basis recorded in the audit trail.

Two distinct procedures operate at two distinct stages and are not interchangeable. Data charting, the transfer of information from each source into the fields of the charting form, is carried out by one reviewer and verified in full against the full text by a second, with the roles alternating across sources. Qualitative coding, the interpretive assignment of charted material to Rodgers’ categories, is carried out independently and in duplicate by two reviewers, as specified in Section 2.9. The two classifications on which the nursing conclusions depend are recorded on the charting form but are interpretive rather than transcriptive and therefore follow the coding procedure and not the charting one. The reviewers will work in two pairs, and all four reviewers will complete a common pilot set of at least five purposively selected sources spanning different cardiovascular diagnoses and evidence types. Disagreements will be reconciled within each pair, issues affecting both teams will be added to the shared codebook, and unresolved conceptual disagreements will be referred to a third reviewer. Agreement on the two classifications on which the nursing conclusions depend, nursing recognizability and source-reported locus of limitation, will be quantified rather than described: both reviewers in each pair code them independently for the pilot set and a further random 20% of included sources, Cohen’s kappa is computed and reported per field and per pair with the number of items coded, and where it falls below 0.70 the rules are revised, the affected sources recoded and both values reported.

The variables to be charted are listed by domain in Table 4 and defined in Supplementary Table S2, which also fixes the recording of source location and of evidential status.

Disciplinary origin is charted as descriptive provenance, not as evidence of ownership: sources are classified as nursing, non-nursing or mixed on journal subject classification, author affiliation and issuing-body context, with the specific field recorded where identifiable and ambiguous sources coded as mixed or unclear and retained.

### 2.8. Critical Appraisal

Consistent with the Joanna Briggs Institute methodology for scoping reviews, methodological quality will not be used as a criterion for study inclusion because the primary aim of this review is conceptual clarification rather than evaluation of intervention effectiveness or estimation of treatment effects. Consequently, no source meeting the predefined eligibility criteria will be excluded solely on the basis of methodological quality.

Nevertheless, the methodological characteristics and evidential status of each included source will be documented and will influence interpretation. A source’s peer-review status alone will not establish quality. The team will distinguish theoretical proposal, empirical observation, guideline or consensus statement, and validated measurement evidence; the rules by which recurrence is counted and by which these categories are weighted are set out in full in Section 2.9.1 and are not repeated here.

This does not exempt the review from appraisal where appraisal is warranted. A definition proposed in a theoretical paper will not be treated as equivalent to a construct examined in primary research, and if the final analysis advances any claim about measurement properties or clinical validity, appropriate appraisal tools will be applied to that subset of sources and reported separately from the conceptual synthesis.

### 2.9. Data Analysis and Synthesis

Data analysis will combine descriptive evidence mapping with directed and inductive qualitative content analysis. Descriptive results will summarize publication and source characteristics, terminology, and approaches used to operationalize exercise intolerance, along the dimensions prespecified in the Secondary Review Questions Section.

Bibliographic records will be managed in Mendeley Reference Manager, descriptive charting in Microsoft Excel, qualitative content analysis in NVivo 15 (Lumivero) and agreement statistics in R (version 4.6.1), with all versions reported. NVivo will hold the coding tree, the coded segments linked to their source locations, the conceptual memos and the audit trail, all of which are exported and deposited as set out in the Data Availability Statement, so that the analysis can be inspected independently of the software.

Rodgers’ evolutionary framework will provide the initial analytical structure, and additional categories will be developed inductively. Two reviewers will independently code the complete eligible corpus, or an explicitly defined and documented portion if a registered amendment is required. A third reviewer will review all contested cases, category definitions and the provisional definition. This is independent double coding, not single primary coding with verification. Coding decisions, disagreements and codebook changes will be documented in the audit trail.

The analysis plan described in this section, including the review questions, the comparative dimensions, the charting variables, the coding framework, the criteria for conclusions and the robustness checks, was developed a priori and registered in the Open Science Framework before screening and data charting commenced. No inferential statistical analysis is planned: quantitative reporting is restricted to descriptive frequencies of source and conceptual characteristics and to inter-rater agreement statistics for calibration. Any deviation from the registered plan will be documented in the amendment log and reported in the completed review.

The analysis will:

− Distinguish surrogate terms from related concepts without assuming conceptual equivalence;
− Distinguish antecedents, indicators and consequences according to their conceptual role within each source;
− Document classification decisions where the same phenomenon may be interpreted differently across studies.

The illustrative exemplar required by Rodgers’ method will be a fictional but clinically credible cardiovascular case, constructed only after the defining attributes have been established and demonstrating each of them explicitly while distinguishing antecedents and consequences from the attributes themselves. It will be presented as an illustration of the literature-derived definition and not as evidence for it, and it will be reviewed by two team members who did not draft it.

#### 2.9.1. Criteria for Conclusions

A candidate defining attribute must be supported by at least two independent conceptually informative sources and must occur in at least two distinct contexts or evidence types, unless a clearly justified rare-context exception is reported. It must describe a characteristic of the concept rather than a cause, indicator or outcome and must assist differentiation from a neighboring concept. Two operational rules govern how recurrence is judged. First, recurrence is counted on charted attribute statements rather than on wording: two sources support the same attribute when their charted statements are judged to express the same characteristic, and the paraphrase linking them is recorded in the codebook, so that verbal repetition of a phrase is neither necessary nor sufficient. Second, sources count as independent only where they do not share an authorship team, do not report the same dataset, cohort or terminology edition, and are not related as an original report and its own derivative: a guideline and the primary study it cites count once, and successive editions of the same terminology count once for the purpose of recurrence, while the differences between those editions are reported separately as evidence of conceptual change. Source credibility is applied as an explicit ordering rather than as a global quality score. Validated measurement evidence and primary empirical studies carry the greatest interpretive weight, followed by guidelines and consensus statements issued by professional bodies, then systematic and scoping reviews, then theoretical and discussion papers, and finally non-peer-reviewed gray literature. An attribute supported only by sources in the last two tiers is reported as tentative and labeled as such in the concept map, and an attribute that loses support when those tiers are removed is reported through the robustness checks specified in Section 2.9.2 rather than carried into the definition. No category will be treated as a subtype unless the original sources explicitly make that classification.

#### 2.9.2. Robustness Checks

Robustness will be examined descriptively by comparing the provisional attributes and definition across peer-reviewed versus gray or non-peer-reviewed sources, empirical versus theoretical or review sources, major cardiovascular diagnoses, acute or post-acute versus chronic contexts, clinical versus rehabilitation or community settings, and earlier versus later publication periods. The team will assess whether each core attribute remains supported when non-peer-reviewed and lower-evidential-status sources are removed.

### 2.10. Reflexivity

Prior to formal analysis, members of the review team will document their assumptions regarding exercise intolerance and its relationship with neighboring constructs, including exercise tolerance, exercise capacity, functional capacity and cardiorespiratory fitness. Sources presenting contradictory, borderline or context-specific conceptualizations will be retained and examined so that the emerging definition reflects the evolutionary nature of the concept rather than reinforcing preconceived assumptions.

The composition of the review team is itself a reflexive consideration. The team is composed of nurse researchers with clinical and research experience in cardiovascular and rehabilitation nursing and in evidence synthesis, analyzing a concept whose literature originates largely in cardiology and exercise physiology. It does not include an exercise physiologist, and the interpretation of physiological material is therefore made from a nursing standpoint. Where a physiological claim is central to a conceptual decision—for example, whether a reported indicator represents integrated oxygen transport or achieved workload—the source wording will be recorded verbatim and the decision flagged for external methodological consultation before it is carried into the synthesis. The disciplinary background of each coder will be reported so that readers can judge the standpoint from which the conceptual definition was developed. Three mitigation procedures follow. Every classification debate that the coding rules do not settle is recorded in the audit trail with the competing readings, the decision and its basis, so that the standpoint from which each judgment was made remains inspectable. A versioned coding decision aid, held with the codebook, carries the operational definitions, the worked examples and the resolved precedents, so that later decisions follow earlier ones. And where a discrepancy turns on the interpretation of physiological material, external colleagues from cardiology, cardiac rehabilitation and exercise physiology are consulted before the decision enters the synthesis, with the question, the response and the resulting decision recorded; these consultations inform classification, are not a consensus procedure and confer no authorship.

### 2.11. Ethics and Dissemination

Ethical approval is not required because this study will analyze publicly available literature and will not involve human participants or identifiable personal data. The protocol has been prospectively registered in the Open Science Framework (OSF), and any important methodological amendments will be documented and reported in the final review.

Findings will be disseminated through publication in a peer-reviewed journal, presentation at national and international scientific meetings, and communication with researchers and healthcare professionals involved in cardiovascular care and rehabilitation.

### 2.12. Methodological Considerations and Anticipated Limitations

Several limitations are anticipated. Conceptual statements may be poorly indexed despite the five databases and gray-literature searching. The evidence base may remain too heterogeneous to support a stable definition or point-of-care nursing operationalization. Language restrictions may under-represent other conceptual traditions. Classifying provenance, attributes, mechanisms, recognizability and actionability involve interpretation, which will be managed through the procedures specified in Section 2.7 and Section 2.9. The output will be a literature-derived clarification, not a validated instrument, diagnostic threshold, professional consensus or device specification. Two further limitations follow from the breadth of the population. Exercise intolerance may not be conceptually equivalent across cardiovascular etiologies: what the term denotes in heart failure with preserved ejection fraction, in heart failure with reduced ejection fraction, in coronary artery disease, in valvular disease and in arrhythmias may differ in mechanism, in empirical referent and in clinical significance. That the locus of limitation varies even within a single diagnostic category is already visible in the literature cited in the Introduction: exercise intolerance in heart failure is described as a cardinal and multidimensional manifestation associated with cardiac reserve, pulmonary and vascular function, skeletal muscle abnormalities, exertional symptoms and prognosis [17], and in heart failure with preserved ejection fraction impaired exercise performance reflects deficits distributed across cardiac, pulmonary, vascular and skeletal muscle reserve rather than a single central abnormality [19]. The breadth of the disease block, widened during revision to include hypertension, cerebrovascular disease, peripheral arterial disease and congenital heart disease, increases this risk rather than reducing it. Aggregating across these categories therefore carries a risk of conceptual dilution, in which a definition broad enough to accommodate every diagnosis ceases to discriminate among any of them. The comparison by diagnosis specified in Section 2.9 and the robustness checks in Section 2.9.2 are the safeguards against this, and where attributes prove to be diagnosis-specific the review will report them as such rather than forcing them into a single definition.

## 3. Expected Outcomes

The review will answer the questions set out in Section 2.2, to the extent that the retrieved literature allows. Those are the questions it will address, not the answers it presumes. Three outcomes are treated as equally admissible: that the retrieved literature supports a stable conceptual definition whose attributes are usable in nursing assessment; that it supports a definition whose attributes are recognizable only through instrumented testing, in which case exercise intolerance is not operationalizable through nursing assessment alone and that will be reported as the finding rather than as a shortfall; and that it does not support a stable definition at all, whether because usage diverges across cardiovascular etiologies, disciplines and time, or because the conceptually informative evidence proves too sparse. None of the statements that follow should be read as a prediction of which of these will obtain.

A literature-derived conceptual definition and concept map will be produced only if the evidence supports sufficient conceptual stability. A null or indeterminate finding will be reported as such and will not be presented as a limitation of the review.

Gaps in conceptual development and inconsistencies in the description of exertional symptoms, activity limitation, physiological capacity and recovery may inform subsequent nursing-sensitive and biosignal-informed research, but this protocol does not test measurement validity, establish clinical thresholds or determine device parameters.

Any measurement framework or device specification will require separate empirical, clinical and consensus work. Formal face and content validation of any resulting conceptual definition and concept map, whether through an external panel of cardiology clinicians, cardiac rehabilitation nurse specialists and exercise physiologists or through a structured member-checking exercise, is identified here as the necessary next step. It is not undertaken within this review because a literature-derived clarification cannot be validated against the same evidence from which it was derived, and because this protocol does not claim professional consensus.

## 4. Conclusions

This protocol describes a nursing-informed, two-phase sequential scoping review and Rodgers’ evolutionary concept analysis designed to clarify how exercise intolerance and activity intolerance are used in adults with cardiovascular disease, particularly during recovery after cardiovascular events. The review will examine conceptual variation, nursing recognizability and actionability using prespecified rules, while allowing for an unstable, null or indeterminate result. It will not claim professional consensus, diagnostic thresholds, measurement validity or device validation.

## Figures and Tables

**Table 1:** Preliminary conceptual boundaries between exercise intolerance and neighboring constructs.

Construct	Provisional Conceptual Boundary	Typical Operational Expression
Exercise intolerance	Limitation or inability to initiate, sustain or recover from a defined exertional demand, integrating physiological reserve, symptoms, safety and performance.	Symptoms, perceived exertion, exercise-test performance, rhythm, hemodynamic responses and recovery.
Cardiorespiratory fitness	Physiological fitness trait reflecting integrated oxygen transport and use, with established prognostic relevance.	Measured VO2max or VO2peak; sometimes estimated in METs.
Exercise capacity	Achieved performance under a specified graded or standardized exercise-test protocol.	Peak workload, exercise duration, METs or CPET variables.
Functional capacity	Ability to perform activities or field-based tasks that approximate everyday function.	Six-minute walk and other functional performance tests.
Exercise tolerance	A term whose use may refer to the ability to initiate, sustain or complete an exertional demand without disproportionate symptoms or physiological instability. This is a sensitizing description, not a review finding.	Workload or duration, symptoms, perceived exertion, physiological responses and recovery.
Activity intolerance	A nursing-oriented term that may describe insufficient capacity to perform or complete a desired activity, expressed through symptoms, signs, response to activity, functional limitation or recovery.	Reported activity performance and completion, exertional symptoms and signs, perceived exertion, vital-sign and rhythm response to activity, and recovery after activity.

Note: The provisional conceptual boundaries in this table are a priori working descriptions adopted to guide, rather than predetermine, screening and data identification. They are not findings of the review, and none is assumed to be correct; each will be revised, replaced or discarded in the light of the charted evidence, and the revision will be reported. Neither walking distance nor peak oxygen uptake alone should be treated as representing the whole construct of exercise tolerance or intolerance. Abbreviations: CPET, cardiopulmonary exercise testing; METs, metabolic equivalents of task; VO2max, maximal oxygen uptake; VO2peak, peak oxygen uptake.

**Table 2:** Population–Concept–Context (PCC) framework and rationale.

Domain	Include	Exclude
Population	Evidence concerning adults aged 18 years or older with cardiovascular disease, including acute, post-acute, rehabilitation and long-term recovery after cardiovascular events. Mixed populations are eligible when cardiovascular data or conceptual use can be separated.	Healthy-only populations; exclusively pediatric populations; non-cardiovascular populations without separable cardiovascular evidence.
Conceptual relevance	Sources that define, characterize, explain, distinguish or explicitly link exercise intolerance, activity intolerance or neighboring construct to one of these focal concepts.	Sources that only report an exercise outcome without conceptual, definitional or interpretive information relevant to the construct.
Context	Any clinical, rehabilitation, community or research setting and any phase of cardiovascular disease or recovery, with post-event recovery charted explicitly.	No contextual exclusion; setting and phase must be charted for comparative analysis.

**Table 3:** Master search strings and their adaptation across databases.

Master Search Strings (PubMed Syntax)
String 1—Focal concept and related nursing concepts: (“exercise intolerance”[tiab] OR “exercise tolerance”[tiab] OR “activity intolerance”[tiab] OR “activity tolerance”[tiab] OR “decreased activity tolerance”[tiab] OR “exertional intolerance”[tiab] OR “exercise limitation”[tiab] OR “exertional limitation”[tiab] OR “Exercise Tolerance”[Mesh])
String 2—Neighboring constructs: (“exercise capacity”[tiab] OR “functional capacity”[tiab] OR “aerobic capacity”[tiab] OR “cardiorespiratory fitness”[tiab] OR “physical endurance”[tiab] OR “physical fitness”[tiab] OR “Physical Endurance”[Mesh] OR “Cardiorespiratory Fitness”[Mesh] OR “Physical Fitness”[Mesh])
String 3—Cardiovascular disease: (“cardiovascular disease”[tiab] OR “cardiovascular diseases”[tiab] OR “heart disease”[tiab] OR “heart diseases”[tiab] OR “cardiac disease”[tiab] OR “cardiac diseases”[tiab] OR “heart failure”[tiab] OR “coronary artery disease”[tiab] OR “coronary heart disease”[tiab] OR “atrial fibrillation”[tiab] OR arrhythmia[tiab] OR “valvular heart disease”[tiab] OR cardiomyopath*[tiab] OR “myocardial infarction”[tiab] OR “ischemic heart disease”[tiab] OR “ischaemic heart disease”[tiab] OR hypertens*[tiab] OR “pulmonary hypertension”[tiab] OR “cerebrovascular disease”[tiab] OR stroke[tiab] OR “peripheral arterial disease”[tiab] OR “peripheral vascular disease”[tiab] OR “congenital heart disease”[tiab] OR “Cardiovascular Diseases”[Mesh] OR “Heart Diseases”[Mesh] OR “Arrhythmias, Cardiac”[Mesh] OR “Hypertension”[Mesh] OR “Stroke”[Mesh] OR “ Hypertension, Pulmonary “[Mesh] OR “Peripheral Arterial Disease”[Mesh] OR “Heart Defects, Congenital”[Mesh] OR “Myocardial Infarction”[Mesh])
String 4—Conceptual framing: (concept*[tiab] OR defin*[tiab] OR taxonom*[tiab] OR terminolog*[tiab] OR nomenclature[tiab] OR construct*[tiab] OR “concept analysis”[tiab] OR “concept clarification”[tiab] OR “Terminology as Topic”[Mesh] OR “Concept Formation”[Mesh])
Combinations: C1 = String 1 AND String 3 AND String 4; C2 = String 2 AND String 3 AND String 4, included only when the neighboring construct is explicitly linked to a focal concept during screening; C3 = String 1 AND String 3 without String 4, run in every database as a primary combination and not as a conditional sensitivity check, and screened by title and abstract rather than by title alone, because conceptual and definitional information is frequently located in the Methods or Results of empirical papers. String 2 is not run without String 4, because the neighboring-construct block combined with the disease block alone returns a set beyond the capacity of the review team, and because a neighboring-construct source is eligible only where it explicitly links that construct to a focal concept, which places it within the reach of C3. All syntax will be translated and reported in full for each interface.

**Table 4:** Preliminary data-charting framework.

Domain	Variables to Chart
Bibliographic profile	Author, year, country, source type, study design, purpose.
Provenance and temporal positioning	Source provenance (nursing; non-nursing; mixed) and the basis on which it was assigned (journal subject classification, author affiliation or nature of the issuing body); publication period relative to the prespecified 2016 cut-off; phase of the cardiovascular pathway.
Population and context	Diagnosis, participant characteristics (age, sex/gender), healthcare setting, disease or recovery phase, exercise modality and relevant contextual characteristics.
Terminology	Focal term, explicit definition, surrogate terms, related concepts, contrasted concepts, theoretical source.
Attributes	Recurrent defining characteristics used to recognize exercise intolerance.
Antecedents	Conditions or events preceding or influencing tolerance: physiological reserve, disease status, treatment, deconditioning, symptoms, and psychological and contextual factors. Direction of each antecedent (predisposing/risk versus protective).
Consequences	Clinical, functional, behavioral and service outcomes associated with the state or change in tolerance.
Operationalization	Exercise task, protocol, intensity, duration, termination criteria, assessment method, measurement instrument, timing and reference standard.
Empirical referents/observed indicators	Performance, symptoms, perceived exertion, ECG/rhythm, heart rate, heart rate variability (time-domain indices such as SDNN and RMSSD, frequency-domain indices such as LF, HF and the LF/HF ratio, and non-linear metrics, each charted as reported), blood pressure, SpO2, respiratory rate, recovery kinetics, movement and posture.
Conceptual interpretation	Dimension represented by each indicator; explicit versus implicit use; contradictions; temporal or contextual variation.
Nursing recognizability and locus of limitation	Whether each defining attribute and empirical referent is: (a) recognizable through routine nursing assessment; (b) requires instrumented testing; (c) supported by both routes; or (d) unclear. Actionability is coded separately only when the source links the referent to a nursing action. Source-reported mechanisms/loci are coded separately from reviewer inference and are not treated as subtypes without explicit source support.

Abbreviations: ECG, electrocardiogram; HF, high-frequency power of heart rate variability; LF, low-frequency power of heart rate variability; LF/HF, ratio of low- to high-frequency power; RMSSD, root mean square of successive differences between normal RR intervals; SDNN, standard deviation of normal-to-normal RR intervals; SpO2, peripheral oxygen saturation measured by pulse oximetry. In this table, LF and HF denote heart rate variability frequency bands and not heart failure.

## Data Availability

The verified charting dataset, data dictionary, Rodgers matrix, codebook, amendment log, audit trail, descriptive tables, robustness-comparison tables and conceptual-model source map will be deposited in the associated OSF project in versioned XLSX, CSV and/or open-text formats. Public files will contain bibliographic metadata, coded conceptual data and only legally permissible short quotations; copyrighted full texts and extensive verbatim extracts will not be shared. Materials will be released under CC BY 4.0, subject to copyright and legal restrictions.

## Supplementary Materials

The following supporting information can be downloaded at https://www.mdpi.com/article/10.3390/nursrep16090323/s1: Checklist S1: The completed PRISMA-P checklist for this protocol [30]; Table S1: The draft and planned search strategies for MEDLINE, CINAHL, Embase, Web of Science, Scopus and gray-literature sources, with interfaces, field codes and planned limits, together with the date and yield of the single MEDLINE feasibility search already performed; no strategy in Table S1 is described as executed until it has been run and its execution date, exact string as run, limits, yield and deduplication record have been entered; Table S2: The preliminary data-charting framework, coding rules and data dictionary.

## Public Involvement Statement

Patient and public involvement was not undertaken in the design of this protocol. No participant consultation is planned before the scoping review and concept analysis are completed. Any subsequent consultation on the comprehensibility of a provisional conceptual definition will be reported separately and will not be presented as professional consensus.

## Guidelines and Standards Statement

This protocol was drafted in accordance with the JBI methodology for scoping reviews [24,25] and will be reported using the PRISMA-ScR [28] and PRISMA-S [29] reporting guidelines; the conceptual analysis follows Rodgers’ evolutionary method [26,27].

## Use of Artificial Intelligence

Generative AI tools were used to support language editing and document organization. The authors reviewed and take responsibility for the final content. ChatGPT (OpenAI ChatGPT 5.6-Sol) and DeepL Translator (SE, 2026) may be used solely to support translation of potentially eligible English-, Portuguese- and Spanish-language records when required. All decision-critical AI-assisted translations will be verified by a reviewer proficient in an eligible language. AI tools will not make eligibility decisions or perform data extraction or conceptual analysis. Tool names, versions, access dates and uses will be reported in the final review. The tools used, their exact versions, the dates of access and the specific sections to which each was applied are recorded in the OSF project and will be reported in full in the completed review, in accordance with ICMJE recommendations on the use of artificial intelligence in scholarly work.

## References

[1] World Health Organization Cardiovascular Diseases (CVDs). https://www.who.int/news-room/fact-sheets/detail/cardiovascular-diseases-(cvds).

[2] Brown T.M., Pack Q.R., Beregg E.A., Brewer L.C., Ford Y.R., Forman D.E., Gathright E.C., Khadanga S., Ozemek C., Thomas R.J. (2025). Core Components of Cardiac Rehabilitation Programs: 2024 Update: A Scientific Statement From the American Heart Association and the American Association of Cardiovascular and Pulmonary Rehabilitation. J. Cardiopulm. Rehabil. Prev..

[3] Mezzani A., Hamm L.F., Jones A.M., McBride P.E., Moholdt T., Stone J.A., Urhausen A., Williams M.A. (2013). Aerobic Exercise Intensity Assessment and Prescription in Cardiac Rehabilitation: A Joint Position Statement of the European Association for Cardiovascular Prevention and Rehabilitation, the American Association of Cardiovascular and Pulmonary Rehabilitation and the Canadian Association of Cardiac Rehabilitation. Eur. J. Prev. Cardiol..

[4] Hansen D., Abreu A., Ambrosetti M., Cornelissen V., Gevaert A., Kemps H., Laukkanen J.A., Pedretti R., Simonenko M., Wilhelm M. (2022). Exercise Intensity Assessment and Prescription in Cardiovascular Rehabilitation and beyond: Why and How: A Position Statement from the Secondary Prevention and Rehabilitation Section of the European Association of Preventive Cardiology. Eur. J. Prev. Cardiol..

[5] Loureiro M., Delgado B., Duarte J., Ângela Ó., Novo A. (2025). A Atividade Física na Pessoa com Intolerância à Atividade [Physical Activity in the Person with Activity Intolerance]. Atividade Física—Um Conceito Central da Enfermagem de Reabilitação.

[6] Cáceres Rivera D.I., Jaimes Rojas L.M., Cristancho Zambrano L.Y., Acosta Barón J.V., Cañon Gómez D.I., Rojas L.Z. (2024). Clinical Validation of the Defining Characteristics of the Nursing Diagnosis “Activity Intolerance” in Patients with Acute Coronary Syndrome. Nurs. Open.

[7] Rodrigues C.G., Moraes M.A., Sauer J.M., Kalil R.A.K., de Souza E.N. (2011). Nursing Diagnosis of Activity Intolerance: Clinical Validation in Patients with Refractory Angina. Int. J. Nurs. Terminol. Classif..

[8] de Souza V., Zeitoun S.S., Lopes C.T., de Oliveira A.P.D., Lopes J.L., de Barros A.L.B.L. (2015). Clinical Usefulness of the Definitions for Defining Characteristics of Activity Intolerance, Excess Fluid Volume and Decreased Cardiac Output in Decompensated Heart Failure: A Descriptive Exploratory Study. J. Clin. Nurs..

[9] Herdman T.H., Kamitsuru S., Lopes C.T. (2024). NANDA International Nursing Diagnoses: Definitions and Classification, 2024–2026.

[10] International Council of Nurses International Classification for Nursing Practice (ICNP). https://www.icn.ch/icnp-browser.

[11] Guazzi M. (2026). Cardiopulmonary Exercise Testing in Contemporary Cardiology: Physiological Insights, Novel Applications and Evolving Algorithms. Heart.

[12] Brazile T.L., Levine B.D., Shafer K.M. (2025). Cardiopulmonary Exercise Testing. NEJM Evid..

[13] Sietsema K.E., Rossiter H.B. (2023). Exercise Physiology and Cardiopulmonary Exercise Testing. Semin. Respir. Crit. Care Med..

[14] Guazzi M., Arena R., Halle M., Piepoli M.F., Myers J., Lavie C.J. (2016). 2016 Focused Update: Clinical Recommendations for Cardiopulmonary Exercise Testing Data Assessment in Specific Patient Populations. Circulation.

[15] Coulshed A., Coulshed D., Pathan F. (2023). Systematic Review of the Use of the 6-Minute Walk Test in Measuring and Improving Prognosis in Patients with Ischemic Heart Disease. CJC Open.

[16] Ross R., Blair S.N., Arena R., Church T.S., Després J.-P., Franklin B.A., Haskell W.L., Kaminsky L.A., Levine B.D., Lavie C.J. (2016). Importance of Assessing Cardiorespiratory Fitness in Clinical Practice: A Case for Fitness as a Clinical Vital Sign: A Scientific Statement from the American Heart Association. Circulation.

[17] Del Buono M.G., Arena R., Borlaug B.A., Carbone S., Canada J.M., Kirkman D.L., Garten R., Rodriguez-Miguelez P., Guazzi M., Lavie C.J. (2019). Exercise Intolerance in Patients With Heart Failure. J. Am. Coll. Cardiol..

[18] Lang J.J., Prince S.A., Merucci K., Cadenas-Sanchez C., Chaput J.-P., Fraser B.J., Manyanga T., McGrath R., Ortega F.B., Singh B. (2024). Cardiorespiratory Fitness Is a Strong and Consistent Predictor of Morbidity and Mortality among Adults: An Overview of Meta-Analyses Representing over 20.9 Million Observations from 199 Unique Cohort Studies. Br. J. Sports Med..

[19] Nayor M., Houstis N.E., Namasivayam M., Rouvina J., Hardin C., Shah R.V., Ho J.E., Malhotra R., Lewis G.D. (2020). Impaired Exercise Tolerance in Heart Failure with Preserved Ejection Fraction. JACC Heart Fail..

[20] Carbogim F.C., Oliveira L.B., Püschel V.A.A. (2016). Critical Thinking: Concept Analysis from the Perspective of Rodger’s Evolutionary Method of Concept Analysis. Rev. Lat. Am. Enfermagem..

[21] Shahsavari H., Zarei M., Aliheydari Mamaghani J. (2019). Transitional Care: Concept Analysis Using Rodgers’ Evolutionary Approach. Int. J. Nurs. Stud..

[22] Liu S., Xiong X.-Y., Chen H., Liu M.-D., Wang Y., Yang Y., Zhang M.-J., Xiang Q. (2023). Transitional Care in Patients with Heart Failure: A Concept Analysis Using Rogers’ Evolutionary Approach. Risk Manag. Healthc. Policy.

[23] Duan X., Ding Y., Ning Y., Luo M. (2024). Application of NANDA-I Nursing Diagnoses, Nursing Interventions Classification, and Nursing Outcomes Classification in Research and Practice of Cardiac Rehabilitation Nursing: A Scoping Review. Int. J. Nurs. Knowl..

[24] Peters M.D.J., Godfrey C., McInerney P., Munn Z., Tricco A.C., Khalil H., Aromataris E., Lockwood C., Porritt K., Pilla B., Jordan Z. (2024). Scoping Reviews. JBI Manual for Evidence Synthesis.

[25] Aromataris E., Lockwood C., Porritt K., Pilla B., Jordan Z. (2024). JBI Manual for Evidence Synthesis.

[26] Rodgers B.L., Knafl K.A. (2000). Concept Development in Nursing: Foundations, Techniques, and Applications.

[27] Tofthagen R., Fagerstrøm L.M. (2010). Rodgers’ Evolutionary Concept Analysis—A Valid Method for Developing Knowledge in Nursing Science. Scand. J. Caring Sci..

[28] Tricco A.C., Lillie E., Zarin W., O’Brien K.K., Colquhoun H., Levac D., Moher D., Peters M.D.J., Horsley T., Weeks L. (2018). PRISMA Extension for Scoping Reviews (PRISMA-ScR): Checklist and Explanation. Ann. Intern. Med..

[29] Rethlefsen M.L., Kirtley S., Waffenschmidt S., Ayala A.P., Moher D., Page M.J., Koffel J.B., Blunt H., Brigham T., Chang S. (2021). PRISMA-S: An Extension to the PRISMA Statement for Reporting Literature Searches in Systematic Reviews. Syst. Rev..

[30] Shamseer L., Moher D., Clarke M., Ghersi D., Liberati A., Petticrew M., Shekelle P., Stewart L.A., PRISMA-P Group (2015). Preferred reporting items for systematic review and meta-analysis protocols (PRISMA-P) 2015: Elaboration and explanation. BMJ.

[31] Fletcher G.F., Ades P.A., Kligfield P., Arena R., Balady G.J., Bittner V.A., Coke L.A., Fleg J.L., Forman D.E., Gerber T.C. (2013). Exercise Standards for Testing and Training: A Scientific Statement from the American Heart Association. Circulation.

[32] Yancy C.W., Jessup M., Bozkurt B., Butler J., Casey D.E., Colvin M.M., Drazner M.H., Filippatos G., Fonarow G.C., Givertz M.M. (2016). 2016 ACC/AHA/HFSA Focused Update on New Pharmacological Therapy for Heart Failure: An Update of the 2013 ACCF/AHA Guideline for the Management of Heart Failure. Circulation.

[33] Haddaway N.R., Page M.J., Pritchard C.C., McGuinness L.A. (2022). PRISMA2020: An R package and Shiny app for producing PRISMA 2020-compliant flow diagrams, with interactivity for optimised digital transparency and Open Synthesis. Campbell Syst. Rev..

