# Supplementary material for: Clarifying Exercise Intolerance in Cardiovascular Disease: Protocol for a Scoping Review and Evolutionary Concept Analysis
Source: Nurs Rep. 2026 Sep 6;16(9):323. doi: 10.3390/nursrep16090323 (PMC13610234; doi:10.3390/nursrep16090323)
Supplement: Supplementary file 1 [file nursrep-16-00323-s001.zip › nursrep-4506190-supplementary.pdf]

Checklist S1. PRISMA-P 2015 checklist

Clarifying Exercise Intolerance in Cardiovascular Disease: Protocol for a Scoping Review and Evolutionary Concept Analysis.

Section numbers and page numbers refer to the revised manuscript submitted with this checklist; section numbers are authoritative and page numbers will be reconfirmed at proof. Because this protocol describes a scoping review with an evolutionary concept analysis rather than a systematic review of intervention effects, four items are reported as adapted or not applicable, with the reason given in each case: item 7 uses the Population–Concept–Context mnemonic in place of PICO, as recommended by JBI for scoping reviews; item 13 refers to conceptual outputs rather than health outcomes; items 15a and 15b do not apply because no quantitative synthesis is planned; and items 16 and 17 are addressed through design and through explicit criteria for conclusions rather than through publication-bias statistics and GRADE. Reporting of the completed review will follow PRISMA-ScR, and reporting of the search will follow PRISMA-S.

Section and topic	Item No	Checklist item	Where reported
**ADMINISTRATIVE INFORMATION**			
Title			
**Identification**	1a	Identify the report as a protocol of a systematic review	Title (p. 1) and article type "Protocol". The title reads "Protocol for a Scoping Review and Evolutionary Concept Analysis"; the abstract and Section 2 identify the design as a scoping review conducted under JBI methodology rather than a systematic review of effectiveness.
**Update**	1b	If the protocol is for an update of a previous systematic review, identify as such	Not applicable. This is not an update of a previous review.
**Registration**	2	If registered, provide the name of the registry (e.g., PROSPERO) and registration number	Abstract, Registration (p. 1): Open Science Framework (OSF) Registries, DOI 10.17605/OSF.IO/7XZ39. Also Ethics and Dissemination, Section 2.11 (p. 15). PROSPERO does not accept scoping reviews, hence OSF.
Authors			
**Contact**	3a	Provide name, institutional affiliation, and e-mail address of all protocol authors; provide physical mailing address of corresponding author	Author list and affiliation (p. 1), including the full postal address of the institution and the e-mail address of every author; corresponding author identified with an asterisk.
**Contributions**	3b	Describe contributions of protocol authors and identify the guarantor of the review	Author Contributions (p. 17), using CRediT-style roles. J.P.S. is identified as guarantor of the review.
**Amendments**	4	If the protocol represents an amendment of a previously completed or published protocol, identify as such and list changes; otherwise, state plan for documenting important protocol amendments	Eligibility Criteria, Section 2.4 (pp. 6–8) states that the extension of the search from MEDLINE alone to five databases and the grey-literature sources is recorded as a documented and dated amendment to the OSF registration. Section 2 (p. 4) and Ethics and Dissemination, Section 2.11 (p. 15) commit to documenting, justifying and reporting any further deviation; Data Availability (p. 17) deposits the amendment log in OSF.
Support			
**Sources**	5a	Indicate sources of financial or other support for the review	Funding (p. 17): the research received no external funding.
**Sponsor**	5b	Provide name for the review funder and/or sponsor	Not applicable. No funder or sponsor; see Funding (p. 17).
**Role of sponsor or funder**	5c	Describe roles of funder(s), sponsor(s), and/or institution(s), if any, in developing the protocol	Not applicable. No funder or sponsor had any role in developing the protocol; see Funding (p. 17).
**INTRODUCTION**			
**Rationale**	6	Describe the rationale for the review in the context of what is already known	Introduction (pp. 1–4), rewritten as five paragraphs and one table: the clinical problem and the point at which nursing meets it; activity

Section and topic	Item No	Checklist item	Where reported
			intolerance as the diagnostic term nursing already holds, and the unresolved question of its relationship to exercise intolerance; the terminological problem itself, demonstrated with three attributed examples; Table 1 (p. 3) on the provisional sensitizing boundaries between exercise intolerance and its neighboring constructs, presented as working distinctions and not as findings; the paragraph setting out the disciplinary gap the review addresses; and the approach with its stated limits.
**Objectives**	7	Provide an explicit statement of the question(s) the review will address with reference to participants, interventions, comparators, and outcomes (PICO)	Aim, Section 2.1 (p. 4) and Review Questions, Section 2.2 (pp. 4–5): one primary and nine secondary questions. As recommended by JBI for scoping reviews, the question is framed with the Population–Concept–Context (PCC) mnemonic rather than PICO, since the review maps conceptual use rather than intervention effects. PCC elements are given in Table 2 (pp. 6–7).
**METHODS**			
**Eligibility criteria**	8	Specify the study characteristics (e.g., PICO, study design, setting, time frame) and report characteristics (e.g., years considered, language, publication status) to be used as criteria for eligibility for the review	Eligibility Criteria, Section 2.4 (pp. 6–8) and Table 2 (pp. 6–7): population, conceptual relevance and context; eligible evidence types, now stated explicitly for each publication type — studies of any design, reviews, clinical practice guidelines, professional position and consensus statements, theoretical and discussion papers, methodological publications, doctoral dissertations and master's theses and institutional reports are eligible; editorials, letters and commentaries only where they advance an explicit definitional or conceptual argument; conference abstracts are not eligible; exclusions; languages English, Portuguese and Spanish; database inception to final search date with no temporal restriction.
**Information sources**	9	Describe all intended information sources (e.g., electronic databases, contact with study authors, trial registers, or other grey literature sources) with planned dates of coverage	Eligibility Criteria, Section 2.4 (pp. 6–8): MEDLINE (PubMed), CINAHL Complete (EBSCOhost), Embase (Ovid), Web of Science Core Collection and Scopus; grey literature in Google Scholar, ProQuest Dissertations & Theses Global and RCAAP; backward and forward citation searching; coverage from inception to the search date, with all sources updated in November 2026. Author contact procedure stated under Search Strategy, Section 2.5 (pp. 8–9). Table S1 carries a full interface-specific strategy and a platform row for every source, including the four-string ProQuest strategy in ProQuest syntax.
**Search strategy**	10	Present draft of search strategy to be used for at least one electronic database, including planned limits, such that it could be repeated	Search Strategy, Section 2.5 (pp. 8–9) and the master search strings in PubMed syntax, Table 3 (p. 9), presented as four separate conceptual strings and three combinations, two of them primary, with the syntax conventions governing truncation, phrase handling and field tags. No limits are applied within the interfaces. Complete planned syntax for all five databases and the grey-literature sources in Table S1; the strategies are described as planned until an execution date, the string as run, the limits, the yield and the export and deduplication record are entered. C1 is the primary focal-concept combination, C2 the neighboring-construct combination, and C3 a further primary combination without the conceptual block, run in every database and screened by title and abstract. PRESS peer review has not yet been carried out and is scheduled, with sentinel-publication testing, to precede execution; the

Section and topic	Item No	Checklist item	Where reported
			procedural rules for the C3 combination — identical two-stage screening, descriptive comparison of C1 and C3 yields per database, and identical inclusion criteria for records unique to C3 — are stated in the same section (p. 8).
Study records			
**Data management**	11a	Describe the mechanism(s) that will be used to manage records and data throughout the review	Study Selection, Section 2.6 (pp. 9–11): records imported into Rayyan for reference management, deduplication and screening. Data Analysis and Synthesis, Section 2.9 (p. 13): Mendeley Reference Manager for bibliographic records, Microsoft Excel for charting, NVivo 15 for coding and audit trail, R for agreement statistics. Data Availability (p. 17): dataset, codebook, matrix and audit trail deposited in OSF.
**Selection process**	11b	State the process that will be used for selecting studies (e.g., two independent reviewers) through each phase of the review (i.e., screening, eligibility, and inclusion in meta-analysis)	Study Selection, Section 2.6 (pp. 9–11): calibration on a common pilot of at least 50 records and 10 full texts with Cohen's and Fleiss' kappa and a stated threshold; two reviewer teams of two, screening titles/abstracts and full texts independently and blinded within Rayyan; disagreements resolved by discussion and third reviewer; one primary exclusion reason recorded per full text; the PRISMA-ScR flow diagram, presented as Figure 1.
**Data collection process**	11c	Describe planned method of extracting data from reports (e.g., piloting forms, done independently, in duplicate), any processes for obtaining and confirming data from investigators	Data Charting, Section 2.7 (pp. 11–12): standardized form pilot-tested by all four reviewers; one reviewer extracts and a second verifies 100% of fields against the full text, roles alternating; disagreements reconciled within pairs and referred to a third reviewer; iterative refinement documented. Agreement on nursing recognizability and on source-reported locus or mechanism is quantified with Cohen's kappa on the pilot set and a further random 20% of included sources, with a 0.70 threshold, recoding where it is not met, and both pre- and post-revision values reported. Author contact procedure under Search Strategy, Section 2.5 (pp. 8–9). Explicit coding rules for the mode-of-recognition classification, with worked examples covering heart rate, blood pressure, SpO_2, ECG findings, exertional symptoms and perceived exertion, and the rule that the classification follows the way the source obtains the referent rather than the parameter itself, are given in Section 2.7 and reproduced in Table S2. Charting (transcriptive) is primary extraction with full verification; qualitative coding (interpretive) is independent and in duplicate, as distinguished in Section 2.7 and specified in Section 2.9. Form, coding rules, worked examples and data dictionary in Table S2.
**Data items**	12	List and define all variables for which data will be sought (e.g., PICO items, funding sources), any pre-planned data assumptions and simplifications	Data Charting, Section 2.7 (pp. 11–12) and Table 4. Assumptions and simplifications stated explicitly: source provenance is recorded as nursing, non-nursing or mixed and is descriptive rather than a comparative dimension; publication period is analyzed against a single prespecified 2016 cut-off (p. 5); source-reported and reviewer-inferred loci of limitation are charted in two separate fields and are never combined in any count; each attribute and referent is coded for whether it is recognizable through nursing assessment, instrumented testing or both, under the coding rules and worked examples reproduced in Table S2, with sources that do not state how a referent was obtained coded as unclear and excluded from the recognizability count. Full data dictionary in Table S2.

Section and topic	Item No	Checklist item	Where reported
**Outcomes and prioritization**	13	List and define all outcomes for which data will be sought, including prioritization of main and additional outcomes, with rationale	Adapted: a scoping review with concept analysis has conceptual outputs rather than health outcomes. The elements sought are defined in Data Charting and Table 4 and prioritized in Expected Outcomes, Section 3 (p. 16): the primary output is a literature-derived conceptual definition with its defining attributes and empirical referents; secondary outputs are the concept map, the delimitation of neighboring constructs and the account of what is recognizable in nursing assessment. Section 3 states that these outputs will be produced only if the evidence supports sufficient conceptual stability, and sets out three equally admissible outcomes: a stable definition usable in nursing assessment; a definition whose attributes are recognizable only through instrumented testing, in which case exercise intolerance is reported as not operationalizable through nursing assessment alone; or no stable definition at all. A null or indeterminate finding is reported as the finding and not as a shortfall of the review. Criteria for Conclusions, Section 2.9.1 (p. 14) sets the evidential threshold for each element.
**Risk of bias in individual studies**	14	Describe anticipated methods for assessing risk of bias of individual studies, including whether this will be done at the outcome or study level, or both; state how this information will be used in data synthesis	Critical Appraisal, Section 2.8 (pp. 12–13): consistent with JBI guidance for scoping reviews, methodological quality is not an inclusion criterion, since the aim is conceptual clarification rather than estimation of effect. The evidential status and source type of every included source are charted and carried into interpretation, and appraisal tools will be applied to any subset about which a claim on measurement properties or clinical validity is advanced. Robustness Checks, Section 2.9.2 (p. 14) test whether core attributes survive removal of lower-evidential-status sources.
Data synthesis			
**Synthesis**	15a	Describe criteria under which study data will be quantitatively synthesized	Not applicable. No quantitative synthesis or meta-analysis is planned; see item 15d.
	15b	If data are appropriate for quantitative synthesis, describe planned summary measures, methods of handling data, and methods of combining data from studies, including any planned exploration of consistency (e.g., I^2, Kendall's tau)	Not applicable. Data are not appropriate for quantitative synthesis; see item 15d.
	15c	Describe any proposed additional analyses (e.g., sensitivity or subgroup analyses, meta-regression)	Robustness Checks, Section 2.9.2 (p. 14): descriptive comparison of the provisional attributes and definition across peer-reviewed versus grey sources, empirical versus theoretical sources, major cardiovascular diagnoses, acute or post-acute versus chronic contexts, care settings, and position relative to the 2016 publication cut-off. Comparative analyses by diagnosis, setting, phase and modality are listed under Data Analysis and Synthesis, Section 2.9 (pp. 13–14).
	15d	If quantitative synthesis is not appropriate, describe the type of summary planned	Data Analysis and Synthesis, Section 2.9 (pp. 13–14): descriptive evidence mapping combined with directed and inductive qualitative content analysis, structured initially by Rodgers' evolutionary categories and extended inductively, with each of the six analytical steps mapped in Section 2.3 to the charting domains that populate it, and the mapping reproduced in Table S2, producing a literature-derived conceptual definition and an integrative concept map. Descriptive results summarize publication characteristics, provenance, diagnosis, setting, phase, evidence type, terminology and operationalization.

Section and topic	Item No	Checklist item	Where reported
**Meta-bias(es)**	16	Specify any planned assessment of meta-bias(es) (e.g., publication bias across studies, selective reporting within studies)	Statistical assessment of publication bias is not applicable because no meta-analysis is planned. Dissemination bias is addressed by design: grey literature, theses, guidelines, terminology standards and institutional reports are eligible and actively sought (pp. 6–8). Robustness Checks, Section 2.9.2 (p. 14) compare peer-reviewed against grey and non-peer-reviewed sources to expose any dependence of the emerging definition on source type.
**Confidence in cumulative evidence**	17	Describe how the strength of the body of evidence will be assessed (e.g., GRADE)	GRADE is not applicable to a conceptual synthesis of heterogeneous source types. Criteria for Conclusions, Section 2.9.1 (p. 14) specifies the evidential requirements for a candidate attribute, antecedent, consequence, empirical referent and surrogate term; it defines how recurrence is counted (on charted attribute statements rather than on wording, with the linking paraphrase recorded in the codebook) and what makes sources independent (no shared authorship team, dataset, cohort or terminology edition, and no original-and-derivative pairing), and applies source credibility as an explicit ordering from validated measurement evidence and primary empirical studies through guidelines and consensus statements, systematic and scoping reviews, and theoretical papers to non-peer-reviewed grey literature; and states that conclusions remain proportional to source credibility and breadth and will not claim consensus, diagnostic thresholds, measurement validity or causal effects. Evidential status is charted for every source (pp. 11–12), examined in Robustness Checks, Section 2.9.2 (p. 14), and the residual interpretive limits are stated under Methodological Considerations and Anticipated Limitations, Section 2.12 (pp. 15–16).

Checklist item wording reproduced from Shamseer, L.; Moher, D.; Clarke, M.; Ghersi, D.; Liberati, A.; Petticrew, M.; Shekelle, P.; Stewart, L.A.; PRISMA-P Group. Pre-ferred reporting items for systematic review and meta-analysis protocols (PRISMA-P) 2015: elaboration and explanation. BMJ. 2015 Jan 2;350:g7647. doi: 10.1136/bmj.g7647. Erratum in: BMJ. 2016 Jul 21;354:i4086. doi: 10.1136/bmj.i4086. PMID: 25555855.

Table S1. Planned search strategies (execution details to be added after the searches are run)

Reported in accordance with PRISMA-S. Every strategy in this table is a planned/draft strategy. The only search already carried out is the MEDLINE (via PubMed) feasibility search of 31 May 2026, which was used to test retrieval and is not part of the review searches. A strategy becomes an executed strategy only once an execution date, the exact string as run, the limits applied, the number retrieved and the export and deduplication record have been entered in this table. No language, date or study-design limits are applied during database searching; eligibility is applied during screening. C1 is the primary focal-concept search. C2 is a neighboring-construct search, and a C2 record is eligible only where the source explicitly defines, distinguishes, interprets or links that construct to exercise intolerance or activity intolerance. C3 is a primary combination and not a conditional sensitivity check: it is run in every database irrespective of the yield of C1, C3 removes the conceptual-framing block (String 4) while retaining the focal-concept block (String 1), so that it retrieves records which name exercise intolerance or activity intolerance in the title or abstract but do not signal conceptual or definitional framing there — characteristically empirical papers whose definition or operationalization of the concept appears in the Methods or Results — rather than leaving them to a contingent check. C3 records are screened by title and abstract, not by title alone. String 2 is not run without String 4, because the neighboring-construct block combined with the disease block alone returns a set beyond the capacity of the review team, and because a neighboring-construct source is eligible only where it explicitly links that construct to a focal concept, which places it within the reach of C3. The number of records contributed by C3, and the number of C3 records ultimately included, will be recorded separately for each platform. Three procedural rules govern C3. Records retrieved by it enter the same two-stage screening as records retrieved by C1 and C2, by title and abstract and then by full text, dually and blinded, against the same eligibility criteria and with no relaxed threshold. The yields of C1 and C3 are compared descriptively for each platform, reporting the number retrieved by each, the overlap, the number unique to C3 and the proportion of each set surviving to full text and to inclusion. And a record identified only by C3 is assessed on exactly the same criteria as any other record, with no additional requirement to display conceptual vocabulary. The PRESS-based peer review has not yet been carried out; it is scheduled to precede execution of the review searches, together with sentinel-publication testing, and any strategy below will be revised and revalidated where a known eligible source is not retrieved.

Search strategies by platform

Platform/interface	Search date/status	Executable strategy
MEDLINE via PubMed	31 May 2026 feasibility; full search pending	S1 Focal concept = ("exercise intolerance"[tiab] OR "exercise tolerance"[tiab] OR "activity intolerance"[tiab] OR "activity tolerance"[tiab] OR "decreased activity tolerance"[tiab] OR "exertional intolerance"[tiab] OR "exercise limitation"[tiab] OR "exertional limitation"[tiab] OR "Exercise Tolerance"[Mesh])<br><br>S2 Neighboring constructs = ("exercise capacity"[tiab] OR "functional capacity"[tiab] OR "aerobic capacity"[tiab] OR "cardiorespiratory fitness"[tiab] OR "physical endurance"[tiab] OR "physical fitness"[tiab]

Platform/interface	Search date/status	Executable strategy
		OR "Physical Endurance"[Mesh] OR "Cardiorespiratory Fitness"[Mesh] OR "Physical Fitness"[Mesh])<br><br>S3 Cardiovascular disease = ("cardiovascular disease"[tiab] OR "cardiovascular diseases"[tiab] OR "heart disease"[tiab] OR "heart diseases"[tiab] OR "cardiac disease"[tiab] OR "cardiac diseases"[tiab] OR "heart failure"[tiab] OR "coronary artery disease"[tiab] OR "coronary heart disease"[tiab] OR "atrial fibrillation"[tiab] OR arrhythmia*[tiab] OR "valvular heart disease"[tiab] OR cardiomyopath*[tiab] OR "myocardial infarction"[tiab] OR "ischemic heart disease"[tiab] OR "ischaemic heart disease"[tiab] OR hypertens*[tiab] OR "pulmonary hypertension"[tiab] OR "cerebrovascular disease"[tiab] OR stroke[tiab] OR "peripheral arterial disease"[tiab] OR "peripheral vascular disease"[tiab] OR "congenital heart disease"[tiab] OR "Cardiovascular Diseases"[Mesh] OR "Heart Diseases"[Mesh] OR "Arrhythmias, Cardiac"[Mesh] OR "Hypertension"[Mesh] OR "Hypertension, Pulmonary"[Mesh] OR "Stroke"[Mesh] OR "Peripheral Arterial Disease"[Mesh] OR "Heart Defects, Congenital"[Mesh] OR "Myocardial Infarction"[Mesh])<br><br>S4 Conceptual framing = (concept*[tiab] OR defin*[tiab] OR taxonom*[tiab] OR terminolog*[tiab] OR nomenclature[tiab] OR construct*[tiab] OR "concept analysis"[tiab] OR "concept clarification"[tiab] OR "Terminology as Topic"[Mesh] OR "Concept Formation"[Mesh])<br><br>C1 = S1 AND S3 AND S4<br><br>C2 = S2 AND S3 AND S4<br><br>C3 (primary combination, run in every database) = S1 AND S3
Embase via Ovid	Planned; update November 2026	S1 Focal concept = (exercise intolerance OR exercise tolerance OR activity intolerance OR activity tolerance OR decreased activity tolerance OR exertional intolerance OR exercise limitation OR exertional limitation).ti,ab. OR exp exercise tolerance/<br><br>S2 Neighboring constructs = (exercise capacity OR functional capacity OR aerobic capacity OR cardiorespiratory fitness OR physical endurance OR physical fitness).ti,ab. OR exp exercise capacity/ OR exp fitness/<br><br>S3 Cardiovascular disease = (cardiovascular disease OR cardiovascular diseases OR heart disease OR heart diseases OR cardiac disease OR cardiac diseases OR heart failure OR coronary artery disease OR coronary heart disease OR atrial fibrillation OR arrhythmia* OR valvular heart disease OR cardiomyopath* OR myocardial infarction OR isch?emic heart disease OR ischaemic heart disease OR hypertens* OR pulmonary hypertension OR cerebrovascular disease OR stroke OR peripheral arterial disease OR

Platform/interface	Search date/status	Executable strategy
		peripheral vascular disease OR congenital heart disease).ti,ab. OR exp cardiovascular disease/ OR exp hypertension/ OR exp cerebrovascular accident/ OR exp peripheral occlusive artery disease/ OR exp congenital heart malformation/ OR exp heart arrhythmia/<br><br>S4 Conceptual framing = (concept* OR defin* OR taxonom* OR terminolog* OR nomenclature OR construct* OR concept analysis OR concept clarification).ti,ab. OR exp nomenclature/<br><br>C1 = S1 AND S3 AND S4<br><br>C2 = S2 AND S3 AND S4<br><br>C3 (primary combination, run in every database) = S1 AND S3
CINAHL Complete via EBSCOhost	Planned; update November 2026	S1 Focal concept = TI ("exercise intolerance" OR "exercise tolerance" OR "activity intolerance" OR "activity tolerance" OR "decreased activity tolerance" OR "exertional intolerance" OR "exercise limitation" OR "exertional limitation") OR AB ("exercise intolerance" OR "exercise tolerance" OR "activity intolerance" OR "activity tolerance" OR "decreased activity tolerance" OR "exertional intolerance" OR "exercise limitation" OR "exertional limitation") OR (MH "Exercise Tolerance+") OR (MH "Activity Intolerance (NANDA)")<br><br>S2 Neighboring constructs = TI ("exercise capacity" OR "functional capacity" OR "aerobic capacity" OR "cardiorespiratory fitness" OR "physical endurance" OR "physical fitness") OR AB ("exercise capacity" OR "functional capacity" OR "aerobic capacity" OR "cardiorespiratory fitness" OR "physical endurance" OR "physical fitness") OR (MH "Physical Fitness+") OR (MH "Physical Endurance")<br><br>S3 Cardiovascular disease = TI ("cardiovascular disease" OR "cardiovascular diseases" OR "heart disease" OR "heart diseases" OR "cardiac disease" OR "cardiac diseases" OR "heart failure" OR "coronary artery disease" OR "coronary heart disease" OR "atrial fibrillation" OR arrhythmia* OR "valvular heart disease" OR cardiomyopath* OR "myocardial infarction" OR "ischemic heart disease" OR "ischaemic heart disease" OR hypertens* OR "pulmonary hypertension" OR "cerebrovascular disease" OR stroke OR "peripheral arterial disease" OR "peripheral vascular disease" OR "congenital heart disease") OR AB ("cardiovascular disease" OR "cardiovascular diseases" OR "heart disease" OR "heart diseases" OR "cardiac disease" OR "cardiac diseases" OR "heart failure" OR "coronary artery disease" OR "coronary heart disease" OR "atrial fibrillation" OR arrhythmia* OR "valvular heart disease" OR cardiomyopath* OR "myocardial infarction" OR "ischemic heart disease" OR "ischaemic heart disease" OR hypertens* OR "pulmonary hypertension" OR "cerebrovascular disease" OR stroke OR "peripheral arterial disease" OR "peripheral vascular disease" OR "congenital heart disease") OR (MH "Cardiovascular Diseases+") OR (MH "Heart Diseases+") OR (MH

Platform/interface	Search date/status	Executable strategy
		"Hypertension+") OR (MH "Stroke+") OR (MH "Peripheral Arterial Disease+") OR (MH "Heart Defects, Congenital+") OR (MH "Arrhythmia+")<br><br>S4 Conceptual framing = TI (concept* OR defin* OR taxonom* OR terminolog* OR nomenclature OR construct* OR "concept analysis" OR "concept clarification") OR AB (concept* OR defin* OR taxonom* OR terminolog* OR nomenclature OR construct* OR "concept analysis" OR "concept clarification") OR (MH "Concept Analysis") OR (MH "Terminology")<br><br>C1 = S1 AND S3 AND S4<br><br>C2 = S2 AND S3 AND S4<br><br>C3 (primary combination, run in every database) = S1 AND S3<br><br>Search modes: Boolean/Phrase; Apply equivalent subjects off. Every CINAHL Subject Heading listed above will be verified against the current CINAHL Headings thesaurus on the day of execution. Any heading that is not present in the thesaurus will be removed and the corresponding free-text terms retained; the substitution will be recorded in the execution log and reported in the completed review.
Scopus	Planned; update November 2026	C1: TITLE-ABS-KEY(("exercise intolerance" OR "exercise tolerance" OR "activity intolerance" OR "activity tolerance" OR "decreased activity tolerance" OR "exertional intolerance" OR "exercise limitation" OR "exertional limitation") AND ("cardiovascular disease*" OR "heart disease*" OR "heart failure" OR "coronary artery disease" OR "coronary heart disease" OR "atrial fibrillation" OR arrhythmia* OR "valvular heart disease" OR cardiomyopath* OR "myocardial infarction" OR "ischemic heart disease" OR "ischaemic heart disease" OR hypertens* OR "pulmonary hypertension" OR "cerebrovascular disease" OR stroke OR "peripheral arterial disease" OR "peripheral vascular disease" OR "congenital heart disease") AND (concept* OR defin* OR taxonom* OR terminolog* OR nomenclature OR construct* OR "concept analysis" OR "concept clarification")). C2: TITLE-ABS-KEY(("exercise capacity" OR "functional capacity" OR "aerobic capacity" OR "cardiorespiratory fitness" OR "physical endurance" OR "physical fitness") AND ("cardiovascular disease*" OR "heart disease*" OR "heart failure" OR "coronary artery disease" OR "coronary heart disease" OR "atrial fibrillation" OR arrhythmia* OR "valvular heart disease" OR cardiomyopath* OR "myocardial infarction" OR "ischemic heart disease" OR "ischaemic heart disease" OR hypertens* OR "pulmonary hypertension" OR "cerebrovascular disease" OR stroke OR "peripheral arterial disease" OR "peripheral vascular disease" OR "congenital heart disease") AND (concept* OR defin* OR taxonom* OR terminolog* OR nomenclature OR construct* OR "concept analysis" OR "concept clarification")). C3 (primary

Platform/interface	Search date/status	Executable strategy
		combination, run in every database): TITLE-ABS-KEY(("exercise intolerance" OR "exercise tolerance" OR "activity intolerance" OR "activity tolerance" OR "decreased activity tolerance" OR "exertional intolerance" OR "exercise limitation" OR "exertional limitation") AND ("cardiovascular disease*" OR "heart disease*" OR "heart failure" OR "coronary artery disease" OR "coronary heart disease" OR "atrial fibrillation" OR arrhythmia* OR "valvular heart disease" OR cardiomyopath* OR "myocardial infarction" OR "ischemic heart disease" OR "ischaemic heart disease" OR hypertens* OR "pulmonary hypertension" OR "cerebrovascular disease" OR stroke OR "peripheral arterial disease" OR "peripheral vascular disease" OR "congenital heart disease")).
Web of Science Core Collection	Planned; update November 2026	C1: TS=(("exercise intolerance" OR "exercise tolerance" OR "activity intolerance" OR "activity tolerance" OR "decreased activity tolerance" OR "exertional intolerance" OR "exercise limitation" OR "exertional limitation") AND ("cardiovascular disease*" OR "heart disease*" OR "heart failure" OR "coronary artery disease" OR "coronary heart disease" OR "atrial fibrillation" OR arrhythmia* OR "valvular heart disease" OR cardiomyopath* OR "myocardial infarction" OR "ischemic heart disease" OR "ischaemic heart disease" OR hypertens* OR "pulmonary hypertension" OR "cerebrovascular disease" OR stroke OR "peripheral arterial disease" OR "peripheral vascular disease" OR "congenital heart disease") AND (concept* OR defin* OR taxonom* OR terminolog* OR nomenclature OR construct* OR "concept analysis" OR "concept clarification")). C2: TS=(("exercise capacity" OR "functional capacity" OR "aerobic capacity" OR "cardiorespiratory fitness" OR "physical endurance" OR "physical fitness") AND ("cardiovascular disease*" OR "heart disease*" OR "heart failure" OR "coronary artery disease" OR "coronary heart disease" OR "atrial fibrillation" OR arrhythmia* OR "valvular heart disease" OR cardiomyopath* OR "myocardial infarction" OR "ischemic heart disease" OR "ischaemic heart disease" OR hypertens* OR "pulmonary hypertension" OR "cerebrovascular disease" OR stroke OR "peripheral arterial disease" OR "peripheral vascular disease" OR "congenital heart disease") AND (concept* OR defin* OR taxonom* OR terminolog* OR nomenclature OR construct* OR "concept analysis" OR "concept clarification")). C3 (primary combination, run in every database): TS=(("exercise intolerance" OR "exercise tolerance" OR "activity intolerance" OR "activity tolerance" OR "decreased activity tolerance" OR "exertional intolerance" OR "exercise limitation" OR "exertional limitation") AND ("cardiovascular disease*" OR "heart disease*" OR "heart failure" OR "coronary artery disease" OR "coronary heart disease" OR "atrial fibrillation" OR arrhythmia* OR "valvular heart disease" OR cardiomyopath* OR "myocardial infarction" OR "ischemic heart disease" OR "ischaemic heart disease" OR hypertens* OR "pulmonary hypertension" OR

Platform/interface	Search date/status	Executable strategy
		"cerebrovascular disease" OR stroke OR "peripheral arterial disease" OR "peripheral vascular disease" OR "congenital heart disease")).
ProQuest Dissertations & Theses Global	Planned; update November 2026	The same four-string architecture as the bibliographic databases, in ProQuest syntax: the field code noft( ) searches all fields except the full text, the asterisk is the truncation operator and multiword expressions are enclosed in double quotation marks. S1 Focal concept = noft("exercise intolerance" OR "exercise tolerance" OR "activity intolerance" OR "activity tolerance" OR "decreased activity tolerance" OR "exertional intolerance" OR "exercise limitation" OR "exertional limitation"). S2 Neighboring constructs = noft("exercise capacity" OR "functional capacity" OR "aerobic capacity" OR "cardiorespiratory fitness" OR "physical endurance" OR "physical fitness"). S3 Cardiovascular disease = noft("cardiovascular disease" OR "cardiovascular diseases" OR "heart disease" OR "heart diseases" OR "cardiac disease" OR "cardiac diseases" OR "heart failure" OR "coronary artery disease" OR "coronary heart disease" OR "atrial fibrillation" OR arrhythmi* OR "valvular heart disease" OR cardiomyopath* OR "myocardial infarction" OR "ischemic heart disease" OR "ischaemic heart disease" OR hypertens* OR "pulmonary hypertension" OR "cerebrovascular disease" OR stroke OR "peripheral arterial disease" OR "peripheral vascular disease" OR "congenital heart disease" OR "cardiac rehabilitation"). S4 Conceptual framing = noft(concept* OR defin* OR taxonom* OR terminolog* OR nomenclature OR construct* OR "concept analysis" OR "concept clarification"). Combinations, entered as saved searches: C1 = S1 AND S3 AND S4; C2 = S2 AND S3 AND S4; C3 = S1 AND S3, screened by title and abstract. No document-type, language or date limit is applied within the interface; eligibility is applied at screening. All records returned by the documented strategies are screened.
Google Scholar	Planned; update November 2026	Google Scholar does not support the master syntax, so the strategy is adapted rather than translated and is reported as run. G1: "exercise intolerance" "cardiovascular disease" (concept OR definition OR terminology). G2: "activity intolerance" (nursing OR cardiac) (concept OR definition OR diagnosis). G3: "exercise tolerance" "heart failure" (concept OR definition). Anywhere-in-article searching; no date limit; sorted by relevance; the first 200 records of each documented search are screened at title and abstract. Date, exact string, number screened and number retained are recorded for each search.
RCAAP	Planned; update November 2026	Portuguese and English searches: ("intolerância ao exercício" OR "tolerância ao exercício" OR "intolerância à atividade" OR "exercise intolerance" OR "exercise tolerance" OR "activity intolerance") AND ("doença cardiovascular" OR "doenças cardiovasculares" OR "insuficiência cardíaca" OR "enfarte do miocárdio" OR "doença cardíaca" OR "cardiovascular disease" OR "heart failure" OR "myocardial infarction") AND (conceito OR definição OR taxonomia

Platform/interface	Search date/status	Executable strategy
		OR terminologia OR constructo OR concept OR definition OR taxonomy OR terminology OR construct).

Execution record to complete after searching

For every platform, the review team will record the exact interface, date and time, search string as executed, limits, number retrieved, export format, deduplication procedure and any translation from the master syntax.

The planned update is November 2026. The final report will distinguish the feasibility search, initial review searches and update searches.

Note on controlled vocabulary. Subject headings are reported exactly as they will be entered. PubMed uses "Hypertension, Pulmonary"[Mesh]; the form "Pulmonary Hypertension" is an entry term and is not the MeSH descriptor. Emtree and CINAHL headings will be confirmed against the current thesauri on the day of execution, and any heading that does not resolve will be dropped in favor of the free-text terms, with the change logged. No block in this table is expressed as a placeholder: every combination is given in the syntax of its own interface and can be pasted and run as printed.

Table S2. Data-charting form, coding rules and data dictionary

Clarifying Exercise Intolerance in Cardiovascular Disease: Protocol for a Scoping Review and Evolutionary Concept Analysis.

This form operationalizes the data-charting framework described under Data Charting, Section 2.7 of the protocol. All four reviewers will complete a common pilot set of at least five purposively selected sources spanning different cardiovascular diagnoses and evidence types. For every included source, one reviewer extracts the data and a second reviewer verifies 100% of fields against the full text; extractor and verifier roles alternate across sources. Reviewers work in two pairs (Joana Pereira Sousa with Maria Loureiro, and Paulo Ferreira with João Graveto). Agreement on the two fields on which the nursing conclusions depend, mode of recognition and Locus of limitation (source-reported), is quantified rather than described: both reviewers in each pair code these fields independently for the pilot set and for a further random 20% of included sources, Cohen's kappa is computed for each field and each pair and reported with the number of items coded, and where kappa falls below 0.70 the coding rules are revised, the affected sources recoded and both the initial and post-revision values reported. Disagreements are reconciled within each pair, issues affecting both teams are added to the shared codebook, and unresolved conceptual disagreements are referred to Paulo Santos-Costa. Charting remains iterative: every change is dated, justified, versioned and retrospectively applied where relevant. Every included source is charted in full; no attribute-saturation or stopping rule is applied.

The Rodgers matrix draws on the Terminology, Rodgers' concept elements, Empirical referents and Conceptual interpretation domains. It arrays attributes, antecedents, consequences, surrogate and related terms, and empirical referents across cardiovascular diagnoses, care settings, phases of the cardiovascular pathway and position relative to the prespecified publication cut-off. Source provenance is carried as a descriptive field so that the relationship between activity intolerance and exercise intolerance can be examined; it is not used to compare disciplines against one another. Direct source statements are distinguished from reviewer interpretation, and the location of conceptually important material is recorded to preserve an auditable link to the source.

Field	Format	Definition / what to record	Allowed values / coding guidance
**Administrative / process**			
Record ID	ID	Unique identifier assigned to each included source.	Auto (e.g., EI-001)
Extractor	Text	Initials of the reviewer who performs primary charting for the source.	J.P.S. / M.L. / P.A.F. / J.G.
Verifier	Text	Initials of the second reviewer who verifies 100% of fields against the full text.	J.P.S. / M.L. / P.A.F. / J.G.
Extraction date	Date	Date the source was charted.	YYYY-MM-DD
Charting version	Text	Version of the charting form used (charting is iterative).	e.g., v1.0, v1.1
Reviewer pair	Categorical	Pair responsible for extraction and verification.	J.P.S.–M.L.; P.A.F.–J.G.
Queries for team	Text	Disagreement or query, resolution within the pair, codebook decision, and referral to P.S.-C. when unresolved.	Free text
**Bibliographic profile**			
First author	Text	Surname of the first author.	Free text
Publication year	Number	Year of publication.	YYYY
Country	Text	Country of the study or corresponding author.	Free text
Source type	Categorical	Type of evidence.	Peer-reviewed empirical; review; theoretical/conceptual; guideline/position statement; consensus document; thesis; book chapter; institutional/grey report

Field	Format	Definition / what to record	Allowed values / coding guidance
Evidential status	Categorical / text	Peer-review and evidential status used to qualify interpretation and robustness checks.	Peer-reviewed vs non-peer-reviewed; empirical vs theoretical/review; design details
Study design	Categorical	Design where applicable.	Cohort; cross-sectional; RCT; qualitative; mixed; N/A
Purpose / aim	Text	Stated aim of the source.	Free text
**Provenance and temporal positioning**			
Source provenance	Categorical	Descriptive provenance of the source, retained so that the relationship between the nursing concept of activity intolerance and the exercise intolerance of the wider clinical literature can be examined. Not a comparative analytic dimension: no comparison between non-nursing disciplines is undertaken.	Nursing; non-nursing; mixed
Basis for provenance	Categorical	Criterion on which provenance was assigned, recorded so the judgment is auditable.	Journal subject classification; author affiliation; nature of issuing body; unresolved
Period relative to cut-off	Categorical	Position of the source relative to the prespecified cut-off anchored on the 2016 statement establishing cardiorespiratory fitness as a clinical vital sign.	Up to and including 2016; 2017 onwards
**Population and context**			
Diagnosis	Categorical	Cardiovascular diagnosis of the population.	Heart failure; CAD; MI; atrial fibrillation; valvular; cardiomyopathy; mixed; other
Index cardiovascular event	Text	Index event, if described.	Free text
Disease severity	Text	Severity descriptor as reported.	e.g., NYHA class; ejection-fraction category
Age	Text	Age of the population.	Mean (SD) or range
Sex / gender	Text	How sex/gender is reported and distribution (SAGER).	% female; terminology used
Care setting	Categorical	Setting of assessment or care.	Coronary care unit; cardiology ward; outpatient; rehabilitation; community
Exercise modality	Categorical / text	Modality of exercise or exertion to which the source refers.	Aerobic; resistance; combined; functional or daily activity; field walking test; not specified
Monitoring level	Categorical	Physiological monitoring during exercise or assessment.	Continuous/telemetry; intermittent; none/unspecified
Disease phase	Categorical	Phase of the cardiovascular pathway.	Acute; early post-acute; stable/chronic
Mobilization stage	Text	Stage of mobilization, if reported.	Free text
Transition	Categorical	Care transition context.	Discharge; rehabilitation transition; N/A
**Terminology**			

Field	Format	Definition / what to record	Allowed values / coding guidance
Focal term used	Text	The exact focal term used in the source.	Free text (verbatim)
Explicit definition	Text	Any explicit definition of the focal term.	Free text; flag Verbatim vs Paraphrased
Source statement	Text	Short verbatim statement supporting a definition or other conceptually important coding.	Verbatim; legally permissible quotation only
Source location	Text	Exact page, section, paragraph, table or figure location of the source statement.	e.g., p. 4, Discussion, para. 2
Reviewer interpretation	Text	Analytical interpretation kept separate from the source statement.	Free text; identify inference explicitly
Surrogate terms	Text	Terms used interchangeably with the focal term.	Free text list
Related concepts	Text	Concepts treated as related but distinct.	Free text list
Contrasted concepts	Text	Concepts explicitly contrasted with the focal term.	Free text list
Relation to activity intolerance	Categorical / text	How the source treats activity intolerance relative to exercise intolerance. Central to the nursing review question.	Used as synonym; used as distinct concept; used without relating the two; not mentioned; free-text note
Terminology source and code	Text	For standardised nursing terminologies, the label as it appears, its diagnostic code, the edition and the date of access. The two terminologies currently diverge — NANDA-I carries decreased activity tolerance, formerly activity intolerance under 00092, while ICNP retains activity intolerance under 10000408 — so the source of each label must be recorded rather than assumed.	NANDA-I (label, code, edition); ICNP (label, code, date of access); not applicable
Theoretical source	Text	Framework or citation underpinning the usage.	Free text / citation
**Rodgers' concept elements**			
Attributes	Text	Necessary or recurring characteristics used to recognize the concept.	Free text
Antecedents	Text	Conditions or events preceding or influencing the concept, with direction.	Physiological reserve; disease status; treatment; deconditioning; symptoms; psychological; contextual. Flag predisposing/risk vs protective
Consequences	Text	Outcomes associated with the state or change in the concept.	Clinical; functional; behavioral; service outcomes
Locus of limitation (source-reported)	Categorical	Locus or mechanism of limitation as explicitly stated by the source. Recorded only where the source itself makes the attribution; where the source states none, the field is	Central/cardiac; peripheral/muscular; ventilatory; symptomatic/perceptual; mixed; not stated. Coded as a source-reported mechanism or classification; never treated as a conceptual subtype

Field	Format	Definition / what to record	Allowed values / coding guidance
		coded Not stated. No reviewer reading is entered here.	unless the source explicitly does so. Agreement on this field is quantified with Cohen's kappa.
Locus of limitation (reviewer-inferred)	Categorical / text	Locus or mechanism the reviewer reads into the source where the source does not state one. Held in a separate field so that it can never be counted as a source attribution, and reported separately from the source-reported field.	Same categories as above, or blank where no inference is made. Never used to claim that the concept has subtypes, never combined with the source-reported field in any count, and reported as reviewer interpretation in the synthesis.
Basis for locus classification	Text	Which of the two locus fields carries the classification and on what basis, recorded so that source attribution and reviewer interpretation remain separable in the audit trail.	Source-attributed (with quoted source wording and location); reviewer-inferred (with the reading and its basis); not classified.
**Operationalization**			
Exercise task / protocol	Text	Exercise task and protocol used to elicit or measure the concept.	Free text
Intensity	Text	Prescribed or achieved intensity.	Free text
Duration	Text	Duration of the task.	Free text
Termination criteria	Text	Criteria for stopping the task.	Free text
Measurement instrument	Text	Instrument or test used.	e.g., CPET; 6-minute walk test; incremental shuttle
Timing	Text	Timing of measurement relative to disease phase.	Free text
Reference standard	Text	Reference standard against which the concept is judged.	Free text
**Empirical referents / observed indicators**			
Empirical referents charted	Multi-select	Observable evidence or operational indicators used to recognize, assess or infer the concept.	Performance; symptoms; RPE; ECG/rhythm; heart rate; heart rate variability; blood pressure; SpO2; respiratory rate; recovery kinetics; movement/posture; other. For heart rate variability, record the index family and the specific index as reported: time domain (e.g., SDNN, RMSSD, pNN50), frequency domain (e.g., LF, HF, LF/HF ratio) or non-linear (e.g., SD1, SD2, sample entropy). Do not convert between families; chart the index the source reports.
Mode of recognition	Categorical	Whether the attribute or referent is recognizable through routine nursing assessment, through instrumented exercise testing, or through both. Routine nursing assessment means observation, history, symptom assessment, perceived exertion, functional observation, vital signs or ordinary bedside monitoring available without specialized exercise-testing	Nursing assessment; instrumented testing; both; unclear

Field	Format	Definition / what to record	Allowed values / coding guidance
		equipment; instrumented testing means CPET, laboratory or field exercise protocols, gas exchange, dedicated sensors or specialized ECG analysis; both requires explicit support for both routes. The classification follows the way the source obtains the referent and not the parameter itself; the worked examples below fix its application to heart rate, blood pressure, SpO2, ECG findings, exertional symptoms and perceived exertion, and are binding on all four reviewers. Agreement on this field is quantified with Cohen's kappa. Determines which elements of the concept are usable at the point of care.	
Actionability	Categorical / text	Whether the source explicitly links the attribute or empirical referent to a nursing action or decision. Coded only where that link is explicit; otherwise the referent is recorded as recognizable but actionability not demonstrated.	Actionable (explicit nursing action link, quote and location recorded); recognizable but actionability not demonstrated; unclear
**Conceptual interpretation**			
Dimension per indicator	Text	Conceptual dimension each indicator is taken to represent.	Free text
Explicit vs implicit use	Categorical	Whether the concept is used explicitly or implicitly.	Explicit; implicit
Contradictions	Text	Internal contradictions or inconsistent usage.	Free text
Temporal / contextual variation	Text	Variation of the concept over time or across care contexts, including earlier versus later sources relative to the 2016 cut-off.	Free text
Boundary or negative case	Text	Contradictory, borderline or negative case that helps delimit the concept.	Free text
Nursing implication	Text	What the source, on this reviewer's reading, implies for how a nurse would recognize or act on exercise intolerance. Kept explicitly separate from the source statement.	Free text; identify inference explicitly
**Robustness**			
Robustness grouping	Multi-select	Groups used in descriptive robustness comparisons.	Peer-reviewed/grey; empirical/theoretical; diagnosis; acute-post-acute/chronic; clinical/rehabilitation/community; period relative to cut-off

Mapping of charting domains onto Rodgers' six analytical steps

Referenced from Section 2.3 of the protocol. Each analytical step is populated from named fields of the charting form above, so that the passage from retrieval to conceptual interpretation is traceable and auditable. No step is populated from the reviewers' recollection of the corpus.

Rodgers' analytical step	Charting domains that populate it
1. Identify the focal concept and associated expressions.	Terminology: focal term, explicit definition, surrogate terms, related concepts, contrasted concepts, relation to activity intolerance, terminology source and code.
2. Define the disciplinary and clinical domains from which evidence is sampled.	Provenance and temporal positioning: source provenance and its basis, period relative to the cut-off. Population and context: diagnosis, index event, severity, age, sex/gender, care setting, exercise modality, monitoring level, disease phase, mobilization stage, transition.
3. Collect evidence on contextual use, defining attributes, antecedents and consequences.	Rodgers' concept elements: attributes, antecedents with direction, consequences. Operationalization: task, protocol, intensity, duration, termination criteria, instrument, timing, reference standard. Empirical referents: indicators charted, with source location.
4. Analyse convergence, divergence, contextual variation and temporal evolution.	Conceptual interpretation: dimension per indicator, explicit versus implicit use, contradictions, temporal and contextual variation, boundary or negative case. Combined with the comparative dimensions prespecified in Section 2.2.1 and the robustness groupings.
5. Construct an illustrative exemplar demonstrating the defining attributes.	The attributes established at step 4, together with the source statements and illustrative material recorded under Rodgers' concept elements and Empirical referents. Constructed only after step 4 is complete.
6. Identify implications, hypotheses and priorities for future refinement.	Mode of recognition, Actionability and Locus of limitation, together with Evidence status and the nursing implication field. These determine what the concept can support at the point of care and what remains for subsequent work.

Reference frames anchoring the Mode of recognition classification

Referenced from Section 2.7 of the protocol. The classification is anchored to these external frames rather than to reviewer judgment alone. Agreement on this field is quantified with Cohen's kappa on the pilot set and a further random 20% of included sources, with a 0.70 threshold.

Route	Reference frame used to delimit it	Examples of charted referents
Routine nursing assessment	The observations and measurements that appear as defining characteristics of activity intolerance in the NANDA-International taxonomy and in the corresponding International Classification for Nursing Practice concepts, together with standard bedside physical assessment parameters. The label, definition, defining characteristics, related factors, associated conditions and diagnostic codes are transcribed verbatim from the editions current at the date of extraction, with the edition and access date recorded, as specified in Section 2.4. This list is fixed before charting begins.	Reported symptoms on exertion; exertional discomfort or dyspnoea; fatigue and generalized weakness; perceived exertion; observed activity performance and completion; vital-sign response to activity obtained by ordinary bedside observation or monitoring; rhythm change observed on routine monitoring; recovery after activity.
Instrumented testing	Procedures that cannot be performed without an exercise-testing laboratory, a standardized exercise protocol, gas exchange analysis, a dedicated sensor or specialized ECG analysis. Delimited by the procedure required, not by the variable produced.	Peak oxygen uptake and other cardiopulmonary exercise testing variables; ventilatory efficiency; achieved workload under a graded protocol; field walking-test distance under a standardized protocol; heart rate variability indices requiring dedicated acquisition; specialized ECG or rhythm analysis.
Both	Explicit support in the source for recognition by each route.	A referent the source describes as observable at the bedside and also as measured under a test protocol; heart rate, blood pressure and SpO_2 fall here where both routes are described.
Unclear	The source does not permit the route to be determined.	Coded unclear; not resolved by reviewer inference.

Worked coding examples for the Mode of recognition classification. Referenced from Section 2.7 of the protocol. These examples are binding on all four reviewers and are held with the coding decision aid. The classification follows the way the source obtains the referent, not the parameter itself. A disagreement is resolved by locating the statement in the source, then by discussion within the pair, then by codebook amendment, and where it remains unresolved by third-reviewer adjudication, with the decision and its basis recorded in the audit trail.

Referent as reported by the source	Coded as	Reason
Heart rate, blood pressure or peripheral oxygen saturation recorded at rest, during ordinary mobilization, or immediately after a walking task.	Routine nursing assessment	Obtained by ordinary bedside observation without specialized equipment or a standardized protocol.
The same parameters recorded during a symptom-limited graded protocol, or reported as a chronotropic index or a heart-rate reserve derived from peak testing.	Instrumented testing	The value exists only as a product of the test protocol and cannot be obtained at the bedside.
Rhythm or ST-segment change read from continuous bedside or telemetry monitoring.	Routine nursing assessment	Ordinary monitoring available to a nurse; no specialized analysis required.
Twelve-lead exercise ECG interpretation; arrhythmia analysis performed within an exercise protocol; heart rate variability indices requiring dedicated acquisition and processing.	Instrumented testing	Requires a standardized protocol, dedicated acquisition or specialized analysis.
Exertional dyspnoea, fatigue, chest discomfort or dizziness; rating of perceived exertion recorded on the Borg scale during ordinary activity.	Routine nursing assessment	Elicited by symptom assessment during usual care.
Rating of perceived exertion recorded at a prescribed workload, or used as a stopping criterion within a standardized protocol.	Instrumented testing	Interpretable only in relation to the protocol that generated it.
Six-minute walk test performed as a standardized protocol on a measured course.	Instrumented testing	Delimited by the procedure required, not by the equipment: a standardized protocol is necessary even though no laboratory is.
The observation that a patient becomes breathless walking the length of a ward corridor.	Routine nursing assessment	Unstandardized clinical observation of activity performance.
A referent the source supports by both routes, or a bedside parameter the source reports as validated against a laboratory measure.	Both	Explicit support in the source for recognition by each route.
The source does not state how the referent was obtained.	Unclear	Not resolved by reviewer inference; does not count towards nursing recognizability.

Abbreviations: CPET, cardiopulmonary exercise testing; ECG, electrocardiogram; HF, high-frequency power of heart rate variability; HRV, heart rate variability; ICNP, International Classification for Nursing Practice; LF, low-frequency power of heart rate variability; LF/HF, ratio of low- to high-frequency power; NANDA-I, NANDA International; RMSSD, root mean square of successive differences between normal RR intervals; RPE, rating of perceived exertion; SDNN, standard deviation of normal-to-normal RR intervals; SpO2, peripheral oxygen saturation measured by pulse oximetry. LF and HF denote heart rate variability frequency bands and not heart failure.
